# Ethics in Physical Medicine and Rehabilitation: Global Research Trends and Thematic Structure—A Bibliometric and Science-Mapping Analysis

**DOI:** 10.3390/healthcare14213754

**Published:** 2026-11-04

**Authors:** Tuba Erdem Sultanoğlu, Hasan Sultanoğlu

**Affiliations:** 1 Department of Physical Medicine and Rehabilitation, Faculty of Medicine, Düzce University, 81620 Düzce, Türkiye; 2 Department of Emergency Medicine, Faculty of Medicine, Düzce University, 81620 Düzce, Türkiye; drsultanoglu@hotmail.com; 3 Department of History of Medicine and Ethics, Institute of Hamidiye Health Sciences, University of Health Sciences, 34668 İstanbul, Türkiye

**Keywords:** physical medicine and rehabilitation, rehabilitation ethics, disability, bibliometric analysis, resource allocation

## Abstract

Background: Ethical issues in physical medicine and rehabilitation (PM&R) span autonomy, disability rights, decision-making, and equitable access. This study mapped the bibliographic and conceptual structure of the ethics-related PM&R literature. Methods: Web of Science Core Collection records published through 2025 were retrieved using PM&R-related terminology and field-restricted ethics terms. The refined search identified 110 records. Following post hoc eligibility reassessment, one veterinary rehabilitation publication was excluded, yielding 109 records for the primary bibliometric analysis. Publication characteristics, citation patterns, and standardized keyword networks were examined. Sensitivity analyses assessed keyword-source selection, keyword-frequency thresholds, and the exclusion of five potentially overlapping publications. Results: The primary dataset comprised 109 indexed records published between 1986 and 2025, including 94 Articles, 12 Reviews, and three records with dual Article/Proceedings Paper classification. These publications received 1918 citations (h-index: 23). The keyword co-occurrence network contained 35 standardized terms and 187 links, forming five thematic communities encompassing care and rehabilitation access, palliative care and participation, informed consent and decision-making capacity, disability and quality, and physicians and AIDS. The sensitivity dataset contained 104 records and 1909 citations, with 33 keyword nodes and 180 links; five thematic communities remained identifiable. Conclusions: The findings characterize recurrent concepts within a defined bibliographic corpus rather than the entire PM&R ethics literature. The broad thematic structure was retained in sensitivity analysis, although publication indicators and detailed network characteristics were affected by potentially overlapping records.

## 1. Introduction

The need for rehabilitation services is increasing globally due to population aging, the growing burden of chronic diseases, and the long-term consequences of trauma and neurological disorders. Studies based on Global Burden of Disease data have shown that a substantial proportion of the world’s population lives with health conditions that may benefit from rehabilitation services at some point during the life course [1]. In this context, Physical Medicine and Rehabilitation (PM&R) is a distinct medical discipline that aims not only to reduce functional loss after disease or injury, but also to preserve independence, social participation, quality of life, and human dignity [2]. Ethical issues in PM&R are related to the classical principles of clinical ethics, yet they arise within a context that differs from acute care settings.

The core principles of biomedical ethics, including autonomy, beneficence, non-maleficence, and justice, must be considered in rehabilitation practice together with long-term functional goals, decision-making capacity, level of dependence, the roles of family members and caregivers, multidisciplinary team decisions, and access to limited rehabilitation resources [3,4]. Therefore, rehabilitation ethics encompasses not only general medical ethics issues such as consent to treatment or treatment refusal, but also PM&R-specific concerns, including patient selection, rehabilitation goal-setting, discharge planning, distribution of responsibilities within the team, and equitable access to rehabilitation services [4,5]. Foundational studies addressing ethical issues in rehabilitation medicine clearly demonstrate the distinctive ethical structure of this field. Blackmer emphasized that the principles of autonomy, beneficence, and justice in rehabilitation medicine are directly related to resource allocation, patient selection, team care, and goal-setting processes [4].

Kirschner et al. reported that ethical issues identified by rehabilitation clinicians were common and were influenced by the dynamic nature of the rehabilitation setting and the team-based model of care [5]. Similarly, studies examining rehabilitation physicians’ attitudes toward medical ethics have shown that resource allocation, patient confidentiality, decision-making capacity, and discharge planning constitute important ethical dilemmas in clinical practice [6]. An important dimension of rehabilitation ethics concerns research and clinical decision-making processes involving vulnerable patient groups and individuals living with disability. In rehabilitation research, issues such as decision-making capacity, communication difficulties, dependency relationships, participant vulnerability, and the assessment of long-term outcomes require particular ethical attention [7].

In clinical practice, ethically complex decisions may arise among physicians, patients, families, and rehabilitation teams in conditions requiring rehabilitation, such as sequelae of cerebrovascular disease, amputation, spinal cord injury, cancer rehabilitation, geriatric rehabilitation, and chronic respiratory failure. These decisions may involve prognosis, patient engagement in treatment, caregiver motivation and support, and the determination of appropriate therapeutic goals [8,9,10]. However, ethics-related publications in PM&R are dispersed across different journals, disciplines, and subthemes. The rehabilitation ethics literature intersects with multiple concepts, including clinical ethics, research ethics, professionalism, disability rights, patient autonomy, shared decision-making, resource allocation, access to resources, and health policy.

This dispersion makes it difficult to comprehensively evaluate the intellectual structure, research trends, and thematic development of the field through traditional narrative reviews alone. Bibliometric analysis and science-mapping methods can systematically reveal the temporal development of a research field, the most productive countries, institutions, authors, and journals, influential publications, and conceptual clusters [11,12,13]. Bibliometric and visualized analysis methods are particularly useful for evaluating the structure of knowledge production in interdisciplinary fields. Techniques such as keyword co-occurrence analysis, co-citation analysis, bibliographic coupling, and collaboration networks make it possible to visualize not only publication output but also the conceptual orientations of a field and their changes over time [11,12,13].

Previous bibliometric research has examined rehabilitation publication patterns broadly, without an ethics-specific focus [14]. More focused reviews have synthesized ethical and bioethical issues in physical therapy [15] and ethical challenges in rehabilitation research [16], using study-level evidence synthesis rather than bibliometric mapping of a PM&R-defined corpus. Building on these complementary approaches, the present study characterizes 109 Web of Science Core Collection records published between 1986 and 2025, retrieved through a PM&R-specific search strategy with field-restricted ethics terminology. Its contribution lies in describing publication, citation, and country-collaboration patterns; identifying five exploratory keyword-based thematic communities; and examining the influence of keyword-source selection and occurrence thresholds on the observed thematic structure. These findings provide a bibliographic characterization of the retrieved literature rather than a comprehensive assessment of rehabilitation ethics or independently validated clinical priorities. By identifying the most productive journals, countries, authors, highly cited publications, and keyword-based thematic clusters, this study provides an overview of how rehabilitation ethics has developed and how it may inform future rehabilitation practice, education, research, and policy.

## 2. Materials and Methods

### 2.1. Study Design and Setting

This study was designed as a bibliometric and visual science-mapping analysis to evaluate global research trends in ethics-related scientific publications within the field of Physical Medicine and Rehabilitation (PM&R). Publication output, citation structure, country, institution, author and journal contributions, and keyword-based conceptual clusters were analyzed. The primary aim was to assess not only the quantitative volume of ethics-related literature in rehabilitation medicine, but also the conceptual structure of its ethical themes and their development over time. The study included Web of Science Core Collection records classified as Articles or Reviews that met the predefined bibliographic search criteria combining PM&R-related terminology with ethics-related concepts. The scope encompassed publications addressing ethical questions directly, as well as publications in which ethically relevant concepts were represented within broader rehabilitation-related research. Accordingly, the dataset was treated as a search-defined corpus of ethics-related PM&R literature rather than as a collection in which ethics was necessarily the primary research objective of every publication. No lower publication year limit was applied, and publications indexed up to the end of 2025 were evaluated. No language restriction was applied. Editorial material, letters, corrections, book chapters, meeting abstracts, and records classified solely as conference proceedings were excluded. Protocol-related phrases were excluded through the database search strategy, as detailed in Section 2.2. The potential implications of this automated exclusion for the retrieval of ethics-focused research protocols are acknowledged as a methodological limitation. Three records were classified by Web of Science as “Article; Proceedings Paper”. These records were retained in the final analysis because they were also indexed as journal articles and met the predefined topic-based eligibility criteria.

### 2.2. Data Source and Search Strategy

Bibliographic data were retrieved from the Web of Science Core Collection (WoSCC). The eligible citation indexes were the Science Citation Index Expanded (SCI-EXPANDED), Social Sciences Citation Index (SSCI), Emerging Sources Citation Index (ESCI), and Arts & Humanities Citation Index (AHCI). Only records classified as Articles or Reviews were considered, and publications from 2026 were excluded to avoid including an incomplete publication year. No language restrictions were applied. The search strategy combined terminology related to Physical Medicine and Rehabilitation (PM&R) with ethics-related concepts. An initial exploratory search used the Topic (TS) field for both concept groups. Because this approach retrieved publications containing generic ethics statements, particularly study protocols, the search strategy was subsequently refined. In the final strategy, PM&R-related terms remained in the Topic field, whereas ethics-related terms were required to occur in the Title (TI), Author Keywords (AK), or Keywords Plus (KP) fields. Records containing the protocol-related expressions “ethics and dissemination”, “study protocol”, “trial protocol”, or “protocol for” were excluded using the Topic field. The complete executable final search strategy, including field tags, Boolean operators, truncation symbols, exclusion clauses, and database restrictions, is provided in Supplementary Methods S1. The executable search query was retrospectively reconstructed from the documented field-level search strategy because the original Web of Science search-history string was not preserved. A record-level consistency check of the archived 110-record dataset was performed using the reconstructed search concepts and available bibliographic metadata. Although the archived records were consistent with the expected search concepts and document-type and publication-year restrictions, this check did not independently establish that re-executing the reconstructed query would reproduce exactly the original retrieval set. The archived bibliographic export is dated 28 May 2026; this date represents the documented export snapshot and should not be interpreted as an independently verified original search date. Citation counts were obtained from this export.

### 2.3. Record Selection and Data Cleaning

**Figure 1:**
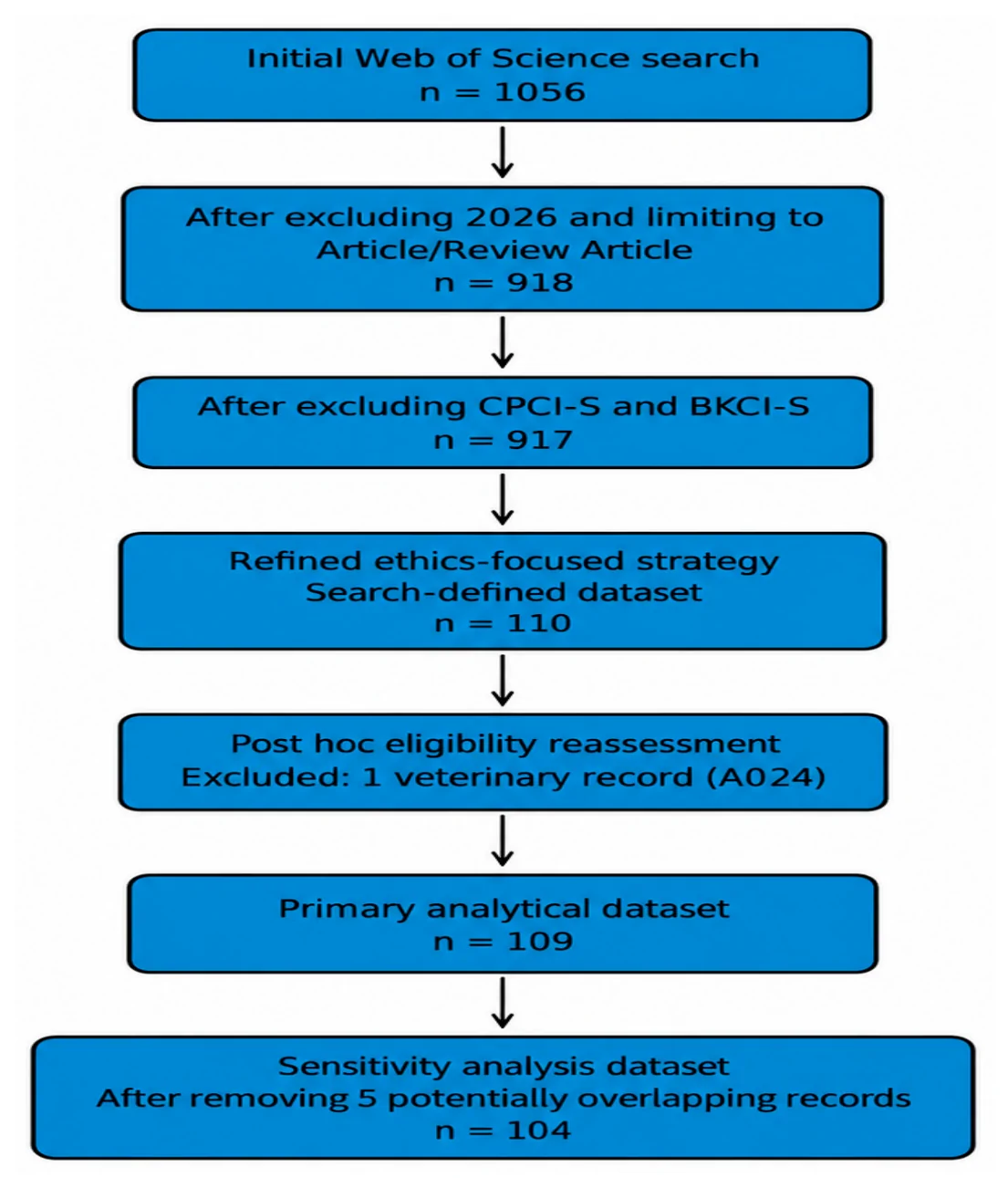
Bibliometric record identification, search refinement, and post hoc eligibility reassessment. The initial Web of Science search yielded 1056 records. After publication-year and document-type restrictions, 918 records remained. Citation-index restrictions further reduced the dataset to 917 records. Refinement of the database search strategy, including field-specific ethics terms and protocol-related exclusions, resulted in 110 records. Subsequent post hoc eligibility reassessment identified and excluded one publication concerning veterinary rehabilitation (A024), resulting in a primary analytical dataset of 109 records. An additional sensitivity analysis was conducted by excluding five potentially overlapping or republished records, yielding a dataset of 104 records. The reduction from 917 to 110 reflects database query refinement rather than individual full-text screening, whereas the subsequent exclusion of A024 was based on substantive eligibility reassessment. The five potentially overlapping records were retained in the primary analysis and excluded only in the sensitivity analysis.

The initial exploratory Web of Science search retrieved 1056 records. After excluding publications from 2026 and restricting document types to Articles and Reviews, 918 records remained. Restriction to the eligible citation indexes further reduced the number to 917 records. The search strategy was then reformulated by requiring ethics-related terms to appear in the Title, Author Keywords, or Keywords Plus fields and by applying the specified protocol-related exclusions. This refined database search yielded 110 records. Following the database search, a post hoc eligibility reassessment was conducted to evaluate the substantive relevance of the retrieved records to ethics in human PM&R. The preliminary examination included publication titles, available abstracts, bibliographic indexing terms, and source information. AI-assisted preliminary checks were used to identify records requiring closer examination. The authors subsequently reviewed 35 flagged publications and considered all of them eligible for inclusion. Eligibility required a meaningful relationship to human rehabilitation practice, services, research, or policy and a substantive ethical dimension. The mere presence of ethics-related indexing terms was not considered sufficient to establish eligibility. One record (A024) concerning veterinary palliative and hospice rehabilitation was excluded because it fell outside the human PM&R context. Consequently, 109 records were retained for the primary bibliometric analysis. The record identification, search refinement, and post hoc eligibility reassessment process is summarized in Figure 1. Importantly, the reduction from 917 to 110 records resulted from modification of the database search criteria rather than the manual exclusion of 807 individual publications following full-text eligibility assessment. Therefore, these records should be interpreted as a bibliographically defined corpus of ethics-related PM&R publications, without assuming that ethics constituted the primary research focus of every included record. No separate systematic title-and-abstract or full-text assessment was undertaken to establish whether ethics constituted the primary research focus of each of the 109 included records; the post hoc reassessment instead evaluated substantive relevance to human PM&R and ethical issues. The final dataset was examined for publication year, document type, language, journal title, author information, institutional affiliations, country information, citation counts, keyword fields, and Web of Science accession numbers. No duplicate Web of Science accession numbers were identified among the 110 initially retrieved records. Following the exclusion of the out-of-scope veterinary publication, the primary analytical dataset comprised 109 distinct indexed records. Nevertheless, distinct indexed records may contain overlapping or republished intellectual content, and this possibility was considered when interpreting publication and author productivity. Five pairs of separately indexed records were identified as potentially overlapping publications because they shared identical titles and author information. Each pair consisted of an earlier publication from 1993–1994 and a corresponding record published in a 1995 supplementary issue of the same journal. We compared publication metadata, available abstracts, and cited references to assess possible republication. Four pairs had identical abstracts after normalization of minor typographical differences, while abstracts were unavailable for both records in the remaining pair. Because separate Web of Science accession numbers do not necessarily represent independent intellectual works, the potential influence of these records was evaluated through a sensitivity analysis in which only the earlier publication from each pair was retained. Author-name variants were additionally examined using available bibliographic information. The variants “Haas JF” and “Haas J” were verified as referring to the same researcher and consolidated under “Haas JF” in the author-productivity analysis. Three records carried the dual Web of Science classification “Article; Proceedings Paper” and were retained because they were also classified as Articles and satisfied the database search criteria. The complete bibliographic list of the 109 records included in the primary analysis is provided in Supplementary File S1.

### 2.4. Bibliometric Analysis

Descriptive bibliometric analyses included annual publication output, publication trends over time, the most productive countries, institutions, authors, and journals, the most highly cited publications, and the most frequently used keywords. Citation analyses were based on the total citation counts provided by Web of Science. The annual distribution of publications was visualized to demonstrate the historical development of the field. Science-mapping analyses included keyword co-occurrence networks, thematic community detection, and country-level collaboration networks. Author collaboration, co-citation, and bibliographic coupling networks were not included in the final analysis; therefore, no conclusions were drawn regarding these network structures. In the keyword analysis, Author Keywords and Keywords Plus were initially evaluated together. To assess the potential influence of combining these two keyword sources, an additional sensitivity analysis was performed using Author Keywords alone. Each standardized term was counted only once per publication, even when it appeared in both keyword fields. Synonymous terms and spelling variants were standardized before analysis. The detailed keyword normalization, term mapping, and exclusion rules are provided in Supplementary Table S1. The field-defining terms “rehabilitation”, “ethics”, “rehabilitation medicine”, and “physical and rehabilitation medicine” were excluded from the keyword-frequency and co-occurrence analyses. For example, “shared decision making” and “shared decision-making” were merged under a single term.

For the revised keyword network analysis, standardized Author Keywords and Keywords Plus were combined at the publication level using binary counting, whereby each keyword and keyword pair were counted at most once per publication. The minimum occurrence threshold was four publications. An undirected co-occurrence network was constructed using Python 3.13.5 (Python Software Foundation, Beaverton, OR, USA) and NetworkX 3.6.1 (NetworkX Developers; https://networkx.org/). Link weights represented the number of publications in which two terms co-occurred. Communities were identified using the weighted Louvain algorithm (resolution = 1; random seed = 42), and the network was visualized using Matplotlib 3.10.8 (Matplotlib Developers; https://matplotlib.org/). Sensitivity analyses were conducted using minimum occurrence thresholds of three and five publications. All terms meeting the specified thresholds were retained, including disconnected terms where applicable.

VOSviewer version 1.6.21 (Centre for Science and Technology Studies, Leiden University, Leiden, The Netherlands) was used for preliminary bibliometric network exploration and visualization [13]. However, the final keyword co-occurrence network and community detection results presented in this study were generated using NetworkX, with visualization performed using Matplotlib, as described above. Additional descriptive bibliometric analyses and thematic mapping were performed using the R-based bibliometrix package and Biblioshiny interface. Bibliometrix is an open-source tool developed for comprehensive science-mapping and bibliometric analysis [12]. The thresholds used for each analysis are reported in the relevant table or figure legend. Country-level collaboration was assessed using institutional affiliation addresses, with each country counted once per publication. Countries appearing together in the same publication were considered connected, and link weights represented the number of shared publications. To examine temporal variation in keyword usage, the mean publication year was calculated for the most frequently occurring standardized keywords. Citation impact was additionally assessed using citations per publication year, calculated by dividing the total citation count by the number of calendar years from publication through 2026, inclusive. Because citations were retrieved on 28 May 2026, this indicator represents an approximate time-adjusted measure rather than a complete calendar-year citation rate. Methodological reporting was also considered in relation to published bibliometric guidance [17].

## 3. Results

The results are presented in two complementary parts. The first summarizes descriptive bibliometric indicators, including publication trends, journal and author productivity, geographic distribution, and citation impact. The second focuses on science-mapping findings, including keyword co-occurrence networks, thematic clusters, country collaboration patterns, and temporal keyword characteristics.

### 3.1. Descriptive Bibliometric Findings

#### 3.1.1. General Bibliometric Characteristics

The revised Web of Science search strategy initially retrieved 110 indexed records meeting the predefined bibliographic criteria. Following post hoc eligibility reassessment, one publication concerning veterinary rehabilitation was excluded, resulting in a primary analytical dataset of 109 records covering 1986–2025. Based on Web of Science document-type classifications, the dataset comprised 94 Articles, 12 Reviews, and 3 records classified as “Article; Proceedings Paper”. Of these publications, 108 were published in English and one in French. The total number of citations was 1918, with a mean of 17.60 citations per publication and a median of 6. The h-index was 23 (Table 1).

#### 3.1.2. Annual Publication Trends

**Figure 2:**
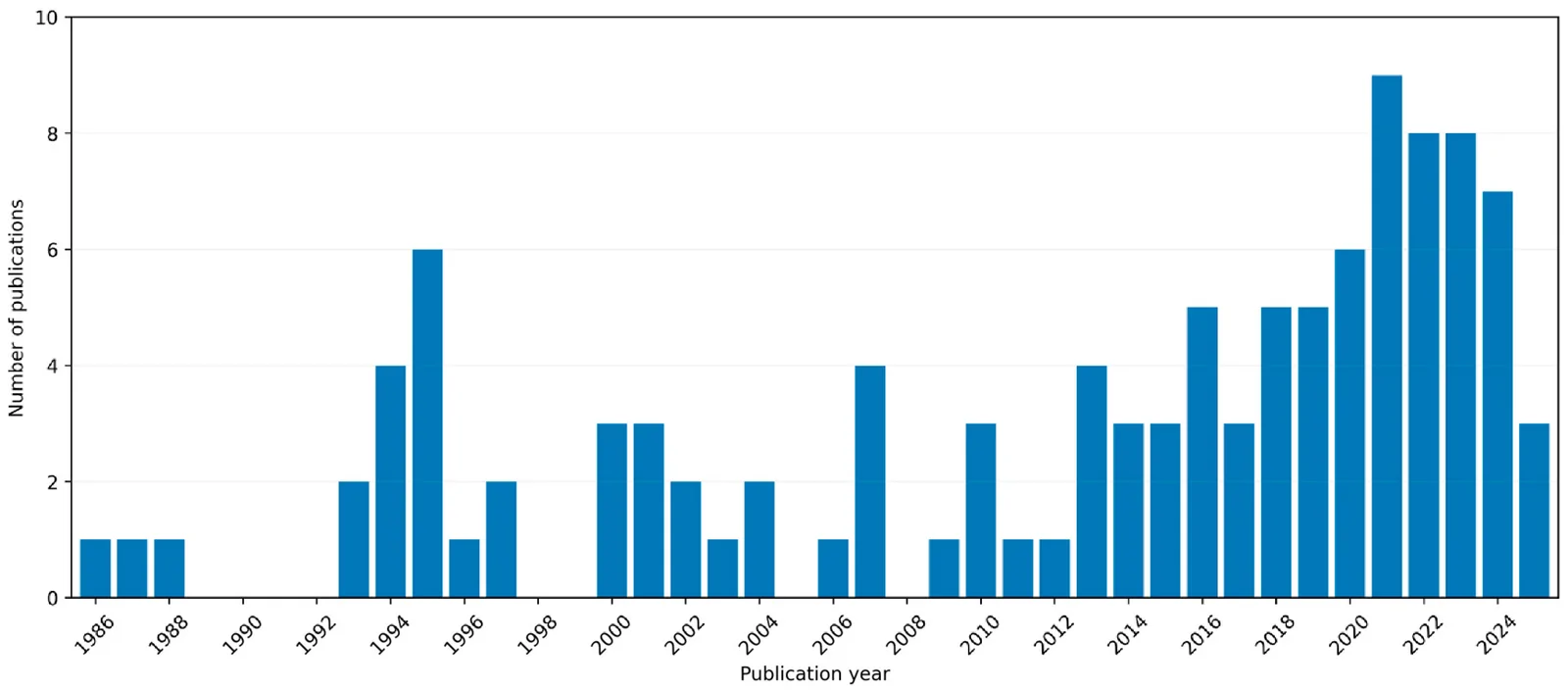
Annual distribution of publications on ethics-related topics in Physical Medicine and Rehabilitation (1986–2025). The figure illustrates the annual number of publications included in the final primary analytical dataset (*n* = 109), following the exclusion of one out-of-scope veterinary publication. The distribution shows a greater concentration of indexed publications in recent years, particularly from 2020 onward.

Ethics-related publications in PM&R were limited in number during the early years. Publications in the 1990s were relatively limited, whereas a greater concentration of records was observed after 2015 within the retrieved dataset. Overall, 56.9% of all publications were published in 2015 or later, and 37.6% were published in 2020 or later. The highest annual number of publications was observed in 2021 (Table 2, Figure 2). An additional temporal analysis was conducted to examine the distribution of frequently occurring keywords across publication years. The mean publication year was 2019.8 for “shared decision making”, 2021.6 for “palliative care”, and 2020.0 for “end of life”. In contrast, “resource allocation” (2007.2), “decision making” (2008.9), and “competence” (2004.3) were associated with earlier mean publication years. These patterns suggest differences in the temporal distribution of ethics-related terminology within the retrieved literature. However, they should not be interpreted as definitive evidence of thematic replacement or increasing relative research interest without comparison against the wider PM&R literature.

#### 3.1.3. Journal-Level Publication Patterns 

The journal with the highest number of publications was Archives of Physical Medicine and Rehabilitation. This journal published 21 articles in the dataset, which received a total of 618 citations. American Journal of Physical Medicine & Rehabilitation ranked second with 19 publications. Together, these two journals accounted for 36.7% of the 109 publications included in the primary analytical dataset (Table 3).

#### 3.1.4. Geographic Distribution and Author Productivity

Among the 98 records for which country information could be identified, the United States was the most productive country, with 50 publications. It was followed by Canada, Italy, Germany, and the United Kingdom. In the country analysis, when a publication included affiliations from multiple countries, each country was counted once for that publication (Table 4). Author productivity was generally low and distributed across multiple researchers. Following bibliographic verification and consolidation of author-name variants, Haas JF (Janet F. Haas) was identified in six indexed records. Other frequently appearing authors contributed three records each. These counts represent indexed publications rather than necessarily distinct intellectual works, as some records may correspond to republications. These counts describe the distribution of indexed author names; network centralization was not evaluated here (Table 4). A supplementary country-level collaboration analysis was conducted using institutional affiliation addresses. Country information was identifiable for 98 of the 109 records. Among these records, 16 publications (16.3%) included affiliations from more than one country. The resulting country co-authorship network comprised 27 countries or regions and 44 distinct international collaboration links. The most frequent bilateral collaboration was between the United States and Canada (*n* = 7). These findings describe international co-authorship within the retrieved dataset rather than the geographical distribution of study participants or research activity beyond Web of Science coverage.

#### 3.1.5. Citation Impact

The most cited publication was the review by Chua on traumatic brain injury rehabilitation. This was followed by publications on a national initiative for cancer rehabilitation, economic and societal issues in spinal cord injury medicine, making patient values visible in health care, and ethical and methodological challenges related to placebo controls in rehabilitation research. The most cited publications addressed topics including patient values, resource allocation, disability, severe illness, end-of-life care, research ethics, and access to rehabilitation (Table 5). Importantly, inclusion in the citation ranking reflects the predefined bibliographic search criteria rather than independent confirmation that ethics was the primary focus of each publication. Accordingly, citation counts should be interpreted as measures of influence within the retrieved corpus, not as rankings of the most influential publications specifically dedicated to rehabilitation ethics. When citation counts were adjusted for publication age, differences in the relative citation visibility of publications became apparent. For example, the publication ranked seventh in Table 5 had 14.00 citations per year, compared with 7.30 citations per year for the top-ranked publication. These findings indicate that total citation rankings and approximate annual citation rates provide complementary perspectives on publication impact.

### 3.2. Science-Mapping Findings

#### 3.2.1. Keyword Analysis and Thematic Structure

The combined Author Keywords and Keywords Plus analysis identified 35 standardized terms occurring in at least four publications after exclusion of field-defining terminology. The most frequent terms were “care” (*n* = 18), “shared decision making” (*n* = 17), “decision making” (*n* = 12), “informed consent” (*n* = 12), “health” (*n* = 11), and “quality” (*n* = 10). More specifically ethics-related terms included “medical ethics” (*n* = 9), “disability” (*n* = 8), “palliative care” (*n* = 8), “competence” (*n* = 6), “end of life” (*n* = 5), and “resource allocation” (*n* = 6) (Table 6).

**Figure 3:**
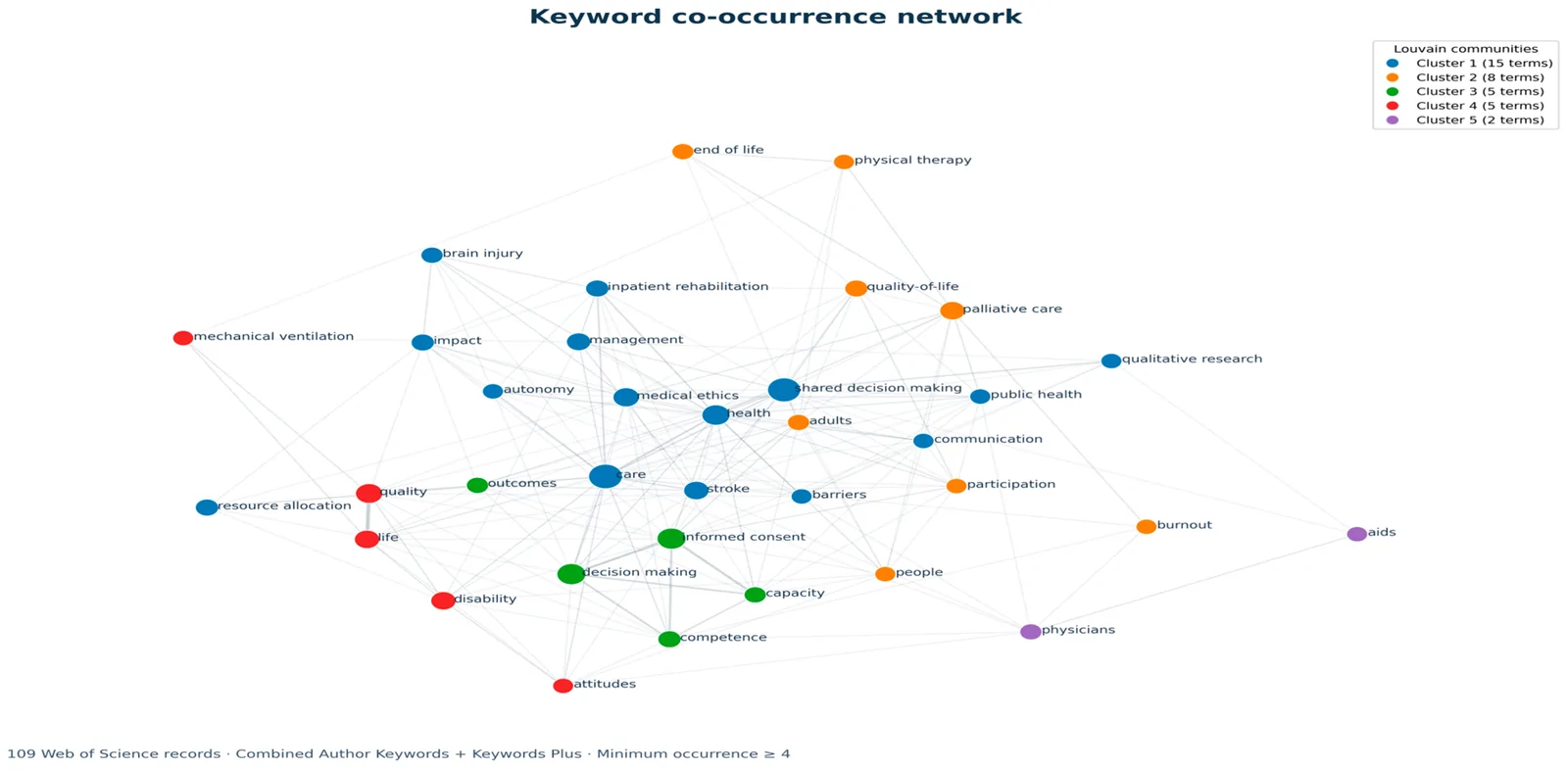
Keyword co-occurrence network. The network presents standardized Author Keywords and Keywords Plus from the 109 Web of Science-indexed records. Keywords occurring in at least four publications were included. Node size represents publication frequency, links indicate keyword co-occurrence, and thicker links reflect greater co-occurrence weight. Node colors represent five communities identified using weighted Louvain clustering. The thematic interpretation of these communities is presented in Table 7.

The keyword co-occurrence network comprised 35 standardized terms forming a connected network with 187 distinct links and a total co-occurrence weight of 276. The Louvain community detection algorithm identified five clusters containing 15, 8, 5, 5, and 2 terms, respectively (Figure 3; Table 7).

The weighted Louvain community detection analysis identified five keyword clusters comprising 15, 8, 5, 5, and 2 terms, respectively. These clusters represented recurring conceptual associations related to care and rehabilitation access, palliative and participation-related concepts, informed consent and decision-making capacity, disability and quality, and physicians and AIDS. The corresponding cluster characteristics, principal standardized keywords, and within-cluster link weights are presented in Table 7. These thematic labels reflect the authors’ interpretation of keyword relationships rather than a qualitative content analysis of the included publications.

Clusters were identified using weighted Louvain community detection in the standardized keyword co-occurrence network derived from the primary analytical dataset (*n* = 109). Terms, *n* indicates the number of standardized keywords assigned to each cluster. Keyword frequencies represent the number of publications containing each term. Within-cluster link weight represents the sum of co-occurrence weights for connections between keywords belonging to the same cluster; connections between different clusters are not included. The principal keywords shown are selected representative terms rather than exhaustive cluster memberships. Interpretive themes were assigned by the authors and should not be regarded as formal classifications of research content.

To evaluate the potential influence of overlapping or republished content, an additional record-level sensitivity analysis was conducted after excluding five potentially overlapping 1995 supplement records. This reduced the dataset from 109 to 104 records. Total citations decreased from 1918 to 1909, while the h-index remained unchanged at 23. The number of publications attributed to the American Journal of Physical Medicine & Rehabilitation decreased from 19 to 14. In the combined keyword co-occurrence analysis, the number of nodes decreased from 35 to 33, the number of distinct links from 187 to 180, and the total link weight from 276 to 258. Five thematic communities were identified in both analyses, although their sizes and keyword memberships differed. These findings suggest that the broad thematic structure remained identifiable after removal of the potentially overlapping records, while some network characteristics and publication productivity indicators were sensitive to their inclusion.

#### 3.2.2. Author Keyword-Only Sensitivity Analysis

To assess the influence of keyword-source selection, an additional analysis was performed using Author Keywords alone. Of the 109 publications included in the primary analysis, 94 contained Author Keywords. After standardization and exclusion of field-defining terminology, 13 terms occurred in at least four publications. The most frequent Author Keywords were “decision making” (*n* = 9), “medical ethics” (*n* = 8), “shared decision making” (*n* = 8), “informed consent” (*n* = 7), “palliative care” (*n* = 6), “end of life” (*n* = 5), and “resource allocation” (*n* = 6). Notably, the general terms “care”, “health”, and “quality”, which were among the most frequent terms in the combined analysis, originated exclusively from Keywords Plus. Their absence from the Author Keyword-only analysis demonstrates that combining the two keyword sources influenced the relative prominence of broad concepts. Nevertheless, decision-making, informed consent, medical ethics, palliative care, and resource allocation remained prominent in the Author Keyword-only analysis. This suggests that several principal conceptual domains were not solely attributable to Keywords Plus, although their relative frequencies and network relationships differed between the two approaches.

#### 3.2.3. Country Collaboration Network

A supplementary country-level collaboration analysis was conducted using institutional affiliation addresses. Country information was identifiable for 98 of the 109 records. Among these records, 16 publications (16.3%) included affiliations from more than one country. The resulting country co-authorship network comprised 27 countries or regions and 44 distinct international collaboration links. The most frequent bilateral collaboration was between the United States and Canada (*n* = 7) (Supplementary Figure S1). These findings describe international co-authorship patterns within the retrieved dataset rather than the geographical distribution of study participants or research activity beyond Web of Science coverage.

#### 3.2.4. Temporal Keyword Patterns

An additional temporal analysis was performed to examine the distribution of frequently occurring keywords across publication years. The mean publication year was 2019.8 for “shared decision making”, 2021.6 for “palliative care”, and 2020.0 for “end of life”. In contrast, “resource allocation” (2007.2), “decision making” (2008.9), and “competence” (2004.3) were associated with earlier mean publication years (Supplementary Figure S2). These findings demonstrate differences in the temporal distribution of ethics-related terminology within the retrieved literature. However, they should not be interpreted as definitive evidence of thematic replacement or increasing relative research interest without comparison against the broader PM&R literature.

## 4. Discussion

This bibliometric and visualized analysis showed that the ethics-related literature in the field of PM&R represents a relatively limited but increasingly visible research area. The final primary dataset included 109 publications published between 1986 and 2025. A substantial proportion of the retrieved records appeared after 2015; however, this concentration does not establish an increase in relative research interest without comparison with overall PM&R publication output. The journals with the highest publication output were Archives of Physical Medicine and Rehabilitation and American Journal of Physical Medicine & Rehabilitation.

At the country level, the United States and Canada were dominant contributors. At the conceptual level, the most frequent standardized terms included “care”, “shared decision making”, “decision making”, “informed consent”, “health”, and “quality”; among the more specifically ethics-related terms, “medical ethics”, “disability”, “palliative care”, “competence”, “end of life”, and “resource allocation” were prominent. These findings indicate that PM&R ethics is not merely the application of general principles of medical ethics to rehabilitation practice, but is shaped by PM&R-specific clinical contexts, including long-term functional goals, disability, patient dependence, multidisciplinary team care, decision-making capacity, fair allocation of resources, and end-of-life care. Therefore, PM&R ethics should be considered a distinct research area at the intersection of clinical ethics, research ethics, disability studies, health policy, and patient-centered care. The increasing publication trend is also consistent with the growing importance of rehabilitation within global health systems.

The World Health Organization’s Rehabilitation 2030 call emphasized the need to integrate rehabilitation into health systems, strengthen service capacity, and expand access to rehabilitation services [18,19]. Similarly, studies based on global burden of disease data have reported that approximately one in three people worldwide lives with a health condition that could benefit from rehabilitation services at some point during the life course [1]. This scale of need requires rehabilitation services to be evaluated not only in terms of clinical effectiveness, but also from an ethical perspective, particularly with regard to equitable access, prioritization, patient selection, and resource allocation. Nevertheless, the refined Web of Science search identified 110 records meeting the specified bibliographic criteria. This figure represents the initial search-defined corpus rather than a comprehensive count of publications primarily focused on ethics in PM&R.

This finding may be interpreted in two ways. First, although ethical issues are frequently encountered in PM&R practice, they may not yet have been sufficiently recognized as an independent research topic. Second, some ethical issues may be discussed under concepts such as “patient-centered care”, “quality of life”, “decision making”, “access to services”, or “professionalism”, without explicitly using the term “ethics”, making them difficult to identify through bibliometric search strategies. Therefore, the current bibliometric visibility of PM&R ethics may underestimate its actual clinical importance.

The sensitivity analysis provided additional insight into the conceptual structure of the retrieved literature. Although the combined keyword analysis emphasized several broad terms, including “care”, “health”, and “quality”, these expressions originated exclusively from Keywords Plus. When the analysis was restricted to Author Keywords, more specific concepts such as decision-making, informed consent, medical ethics, palliative care, and resource allocation remained prominent. This finding indicates that the choice of keyword source affects the relative visibility of thematic concepts. Therefore, the combined analysis should be interpreted as a broad bibliographic representation of the literature rather than a direct reflection of the ethical priorities explicitly identified by the authors of the included studies.

The cluster-based analysis provided a more detailed perspective on the conceptual relationships within the retrieved literature. The identified communities broadly reflected decision-making and rehabilitation care, palliative and end-of-life considerations, informed consent and decision-making capacity, and disability-related concepts. These patterns suggest that ethics-related terminology in PM&R is distributed across several interconnected clinical and social contexts rather than forming a single homogeneous research theme. However, the clusters should be interpreted as bibliographic associations rather than definitive classifications of ethical research domains. The relatively small dataset, the influence of Keywords Plus, and the variation in network composition across occurrence thresholds should be considered when interpreting these findings.

The prominence of “shared decision making”, “decision making”, “informed consent”, “competence”, and “autonomy” in the keyword analysis indicates that patient autonomy and decision-making processes are central to PM&R ethics. However, autonomy in the rehabilitation context differs from isolated treatment decisions commonly encountered in acute care settings. Rehabilitation is a long-term, goal-oriented, team-based process that often requires the active participation of family members or caregivers. Therefore, the patient’s decision-making capacity, understanding of functional goals, motivation, communication skills, psychosocial status, and care environment directly influence ethical decision-making processes.

The person-centered rehabilitation model emphasizes that rehabilitation should be structured around patients’ values, life roles, goals, and expectations for participation [20]. This approach indicates that autonomy in PM&R ethics is not limited to “giving consent” or “refusing treatment”; rather, it requires a relational decision-making process that takes into account patients’ meaningful life goals, functional priorities, and social context. Similarly, the shared decision-making model has been defined as a staged communication process in which clinicians and patients discuss available options, possible benefit–harm balances, and decisions aligned with patient preferences [21]. In PM&R practice, this model is particularly important in ethically sensitive areas such as determining the level of amputation, admission to intensive rehabilitation programs, discharge planning, use of assistive devices, and defining rehabilitation goals in advanced illness.

A rehabilitation-specific patient-centered ethics analysis model also argues that ethical decisions should not be based solely on abstract principles, but should be evaluated together with the patient’s functional status, environmental context, team structure, and rehabilitation goals [22]. This perspective is consistent with the thematic distribution observed in our study. Indeed, the prominent keywords encompass patient-centered goal-setting, shared decision-making, and ethically sensitive evaluation of functional outcomes. The prominence of “resource allocation” and “inpatient rehabilitation” in our study highlights the central role of justice in PM&R ethics.

In many health systems, rehabilitation services are delivered under constraints related to bed capacity, workforce, equipment, financing, and time. Therefore, decisions about who should be admitted to intensive rehabilitation, which patients should be prioritized, how potential functional gain should be assessed, and how socially disadvantaged patients should be protected within this process represent important ethical questions.

Studies on admission decisions to rehabilitation units indicate that patient selection cannot be reduced solely to medical prognosis or expected functional gain [9]. Expected benefit should be considered together with patient need, equitable access, prevention of discrimination, lack of social support, and vulnerability. Otherwise, directing rehabilitation resources only toward patients with the highest potential for functional improvement may systematically exclude those with severe disability, limited social support, or complex needs. This creates a PM&R-specific ethical tension between the principles of beneficence and justice.

In this respect, the findings of our study suggest that PM&R ethics research should address not only the question of “who benefits most from rehabilitation?” but also “who can access rehabilitation fairly?” This point is particularly important in the context of medical ethics, because access to rehabilitation services is not merely a matter of clinical service planning, but also an ethical issue related to the right to health, disability justice, and the equitable distribution of resources.

The visibility of “disability” and related concepts in the keyword analysis indicates that PM&R ethics is directly connected to disability rights and health equity. PM&R physicians and rehabilitation teams play a central role in the care of individuals living with disability. Therefore, PM&R ethics should encompass not only the individual patient–physician relationship, but also societal prejudices toward disability, barriers to health care access, physical and communicative accommodations, and risks of institutional discrimination.

Biases, inadequate accessibility arrangements, and communication barriers encountered by individuals living with disability in health care settings have been identified in the literature as important sources of health inequity [23,24]. In this context, PM&R ethics requires an ethical framework that does not view disability merely as a functional impairment to be treated, but instead takes into account individuals’ rights, participation, self-determination, and social position. In our study, the co-occurrence of concepts such as disability, shared decision making, and patient autonomy is consistent with the presence of rights-based and patient-centered themes in the retrieved literature; it does not establish a temporal shift.

The prominence of publications related to “palliative care”, “end of life”, and advanced illness in our findings indicates that rehabilitation is not limited to recovery and functional improvement goals alone. In conditions such as advanced cancer, severe neurological injury, spinal cord injury, lower-limb amputation, and chronic respiratory failure, the aims of rehabilitation may sometimes involve improving functional independence, whereas in other cases they may involve symptom control, reducing caregiver burden, supporting safe discharge, improving communication, and maintaining quality of life.

Decisions regarding referral to and admission for rehabilitation services in patients with advanced cancer raise ethical questions about how prognosis is assessed, how realistic patients’ expectations are, and how rehabilitation is aligned with overall goals of care [8,25]. Similarly, mortality conversations and decision-making processes before amputation are ethically important in terms of enabling patients to understand risks, express their preferences, and allowing clinicians to communicate uncertainties transparently [26]. These findings suggest that end-of-life care in the PM&R ethics literature is not solely a topic belonging to palliative medicine, but constitutes a specific ethical domain in which rehabilitation goals are redefined, patient and family expectations are addressed, and the meaning of care is reconsidered.

The United States and Canada accounted for the largest numbers of publications among records with available country information. However, this geographical distribution reflects the representation of institutional affiliations within the retrieved Web of Science corpus rather than the overall volume or institutional development of rehabilitation ethics research worldwide. The observed differences may be influenced by database coverage, indexing practices, language-related factors, and incomplete affiliation information. Therefore, these findings do not allow conclusions regarding the degree of institutionalization or research capacity in low- and middle-income countries. Nevertheless, variations in rehabilitation resources, health care systems, social policies, and cultural contexts underscore the importance of examining ethical issues across diverse geographical settings. Future studies incorporating additional bibliographic databases and regionally representative sources may provide a more comprehensive understanding of geographical patterns in PM&R ethics research.

One of the important methodological findings of this study is that ethics-related terms may easily produce misleading results in broad bibliometric searches. In the initial broad search, many study protocols were retrieved solely because they contained standard phrases such as “ethics and dissemination”. This indicates that bibliometric studies on ethics based only on broad terms such as “ethic*” may capture publications without substantive ethical content. Therefore, in the final analysis, ethics-related terms were required to appear in the title, author keywords, or Keywords Plus fields, and standard ethics statement phrases commonly found in study protocols were excluded. Although this approach reduced the number of retrieved records, the reduction reflected changes in the bibliographic search criteria rather than independent verification of substantive ethical relevance. Consequently, the refined search cannot be assumed to have eliminated all irrelevant publications or to have identified exclusively ethics-focused research. In bibliometric analyses, large datasets may appear attractive; however, in studies of ethics, the conceptual specificity of the dataset is at least as important as the number of publications included.

The identified keyword patterns may provide a preliminary framework for developing case-based ethics education in PM&R. In particular, the frequent occurrence of “shared decision making”, “informed consent”, “decision making”, and “competence” suggests that educational modules could address patient autonomy, assessment of decision-making capacity, and communication during rehabilitation goal-setting. The thematic associations involving “resource allocation” and “disability” could inform clinical discussions about equitable access to rehabilitation services and fair patient selection, whereas the prominence of “palliative care” and “end of life” could support educational scenarios concerning rehabilitation goals in advanced illness. These applications represent potential educational uses of the bibliometric findings rather than empirically validated curriculum priorities.

For future research planning, the identified thematic associations may help generate research questions concerning patient and family participation, ethical decision-making within rehabilitation teams, equitable resource allocation, disability-related barriers, and rehabilitation goals in advanced illness; however, these topics should be regarded as potential directions for investigation rather than demonstrated gaps in the existing literature. The present bibliometric analysis did not classify all records by empirical study design and therefore cannot quantify the balance between conceptual and empirical research. In particular, studies incorporating patient and family perspectives, research evaluating ethical conflicts within rehabilitation teams, resource allocation models, educational interventions aimed at reducing disability-based discrimination, and investigations into the ethical implications of emerging technologies such as telerehabilitation, robotic rehabilitation, and artificial intelligence represent important areas for future research.

One of the strengths of this study is its conceptually focused search strategy, which directly targeted the ethics literature in PM&R and reduced contamination from non-relevant records. In addition, the study evaluated not only descriptive bibliometric indicators, but also keyword-based thematic structure and science mapping. Thus, both the quantitative and conceptual landscape of the PM&R ethics literature was demonstrated.

This study has several limitations. First, the analysis was restricted to the Web of Science Core Collection, and the findings should not be considered representative of all global research on rehabilitation ethics. Second, the search strategy depended on selected PM&R-related and ethics-related terms, potentially excluding relevant interdisciplinary publications and studies that addressed ethical issues without explicitly using these terms. An additional limitation concerns the retrospective reconstruction of the final Web of Science search query. Although the archived records were checked for consistency with the documented search concepts and bibliographic restrictions, the original saved search-history string was unavailable. Consequently, exact reproduction of the historical retrieval set using the reconstructed query could not be independently established. This limitation should be considered when interpreting the reproducibility and completeness of the bibliographic corpus. Furthermore, although restricting ethics-related terms to the title, Author Keywords, or Keywords Plus fields improved bibliographic specificity, the presence of such terms does not necessarily establish that ethics was the primary focus of an individual publication. Third, combining Author Keywords and Keywords Plus may have increased the representation of broad concepts because these keyword sources are generated differently. Fourth, the exclusion of protocol-related expressions, editorials, letters, and other document types may have omitted relevant contributions to ethical debates. Fifth, country information was unavailable for 11 records, and affiliation-based geographic attribution does not necessarily represent the locations of study participants or the distribution of ethics scholarship. Incomplete author-name disambiguation and potentially overlapping or republished material may also influence productivity and citation indicators. Finally, the concentration of publications in recent years does not independently demonstrate increasing interest relative to the overall growth of PM&R literature. The identified thematic patterns and apparent research gaps should therefore be interpreted within the limitations of database coverage, retrieval criteria, and bibliometric methodology, rather than as comprehensive assessments of clinical or ethical priorities.

## 5. Conclusions

This bibliometric analysis examined 109 Web of Science-indexed records retrieved using PM&R-related and ethics-related search terms, covering the period from 1986 to 2025. These findings reflect the bibliometric visibility of ethics-related concepts within the retrieved literature and should not be interpreted as an exhaustive representation of rehabilitation ethics scholarship. Publication output was concentrated in recent years and in North America. Decision-making, disability, palliative care, and resource allocation were prominent keyword themes. These findings describe the retrieved literature and may inform future empirical research and ethics education; they do not establish clinical priorities or treatment effects.

## Figures and Tables

**Table 1:** General bibliometric characteristics of the dataset.

Characteristic	Value
Total number of publications	109
Publication year range	1986–2025
Number of journals/sources	51
Number of indexed author-name strings	481
Number of institutions	227
Number of countries/regions	27
Records with identifiable country/region information	98
Total number of citations	1918
Mean citations per publication	17.60
Median citations	6
h-index	23
Articles	94
Reviews	12
Article; Proceedings Paper	3
Publications in English	108
Publications in French	1
Publications published in 2015 or later	62
Publications published in 2020 or later	41

Citation counts refer to the 28 May 2026 export. Countries/regions were counted from affiliation address locations, with England and Scotland grouped as the United Kingdom. Author-name strings have not been fully disambiguated.

**Table 2:** Annual distribution of publications and citations.

Year	Number of Publications	Total Citations	Year	Number of Publications	Total Citations
1986	1	21	2010	3	123
1987	1	39	2011	1	3
1988	1	37	2012	1	39
1993	2	34	2013	4	60
1994	4	61	2014	3	55
1995	6	13	2015	3	33
1996	1	3	2016	5	261
1997	2	13	2017	3	40
2000	3	48	2018	5	33
2001	3	32	2019	5	34
2002	2	60	2020	6	203
2003	1	9	2021	9	112
2004	2	13	2022	8	148
2006	1	2	2023	8	48
2007	4	258	2024	7	32
2009	1	48	2025	3	3

Note: Annual publication counts represent the number of records published in each year. Total citations represent the cumulative Web of Science Core Collection citation counts of publications from the corresponding year, as recorded in the bibliographic export dated 28 May 2026. They do not represent citations received during that calendar year.

**Table 3:** Most productive journals.

Journal	Records, *n*	Total Citations	Percentage (%)
Archives of Physical Medicine and Rehabilitation	21	618	19.3
American Journal of Physical Medicine & Rehabilitation	19	232	17.4
BMJ Open	7	211	6.4
Archives of Rehabilitation Research and Clinical Translation	4	49	3.7
Journal of Head Trauma Rehabilitation	3	18	2.8
Journal of Rehabilitation Medicine	2	29	1.8
Romanian Journal of Legal Medicine	2	2	1.8
BMC Musculoskeletal Disorders	2	41	1.8
Current Physical Medicine and Rehabilitation Reports	2	8	1.8
Spine	2	85	1.8

Percentages use 109 records as the denominator. Journals tied at two publications are selectively displayed; this is not an exhaustive list of all tied sources.

**Table 4:** Most productive countries/regions and authors.

Category	Country/Region or Author	Number of Publications
Country/region	United States	50
Country/region	Canada	17
Country/region	Italy	8
Country/region	Germany	5
Country/region	United Kingdom	5
Country/region	Denmark	4
Country/region	New Zealand	4
Country/region	Japan	3
Country/region	China	2
Country/region	France	2
Country/region	Iran	2
Country/region	Israel	2
Country/region	Malaysia	2
Country/region	Netherlands	2
Country/region	Norway	2
Country/region	Romania	2
Country/region	Sweden	2
Country/region	Taiwan	2
Author	Haas JF	6
Author	Blackmer J	3
Author	Friedman A	3
Author	Giardini A	3
Author	Maffoni M	3
Author	Setti I	3
Author	Sommovigo V	3
Author	Constantinovici M	2
Author	Leonard C	2
Author	Nica AS	2
Author	Sayre G	2
Author	Strax TE	2
Author	Treger I	2
Author	Turner AP	2

Each country/region was counted once per record using the institutional affiliation addresses; country information was available for 98 of the 109 records included in the primary analytical dataset. Country and author entries are arranged in descending order of publication count, with alphabetical ordering for ties. Author rows show selected indexed names; ties are not exhaustive. Author-name variants were standardized where bibliographic identity could be verified. “Haas JF” and “Haas J” were consolidated as “Haas JF” based on bibliographic identity verification. Productivity counts represent indexed records and may include separately indexed republications.

**Table 5:** Most cited publications.

Rank	First Author	Year	Publication Title	Journal	Citations	Citations per Year
1	Chua KSG	2007	A brief review of traumatic brain injury rehabilitation	Annals Academy of Medicine Singapore	146	7.30
2	Stout NL	2016	Toward a National Initiative in Cancer Rehabilitation: Recommendations From a Subject Matter Expert Group	Archives of Physical Medicine and Rehabilitation	145	13.18
3	Priebe MM	2007	Spinal cord injury medicine. 6. Economic and societal issues in spinal cord injury	Archives of Physical Medicine and Rehabilitation	96	4.80
4	Mangin D	2016	Making patient values visible in healthcare: a systematic review of tools to assess patient treatment priorities and preferences in the context of multimorbidity	BMJ Open	95	8.64
5	Fregni F	2010	Challenges and Recommendations for Placebo Controls in Randomized Trials in Physical and Rehabilitation Medicine A Report of the International Placebo Symposium Working Group	American Journal of Physical Medicine & Rehabilitation	95	5.59
6	Hvidt NC	2020	What is spiritual care? Professional perspectives on the concept of spiritual care identified through group concept mapping	BMJ Open	77	11.00
7	Jesus TS	2022	Person-Centered Rehabilitation Model: Framing the Concept and Practice of Person-Centered Adult Physical Rehabilitation Based on a Scoping Review and Thematic Analysis of the Literature	Archives of Physical Medicine and Rehabilitation	70	14.00
8	Hardart MKM	2002	Respiratory support in spinal muscular atrophy type I: A survey of physician practices and attitudes	Pediatrics	57	2.28
9	Schönhofer B	2021	Prolonged Weaning: S2k Guideline Published by the German Respiratory Society	Respiration	48	8.00
10	Tamburrini G	2009	Brain to Computer Communication: Ethical Perspectives on Interaction Models	Neuroethics	48	2.67

Citations refer to the 28 May 2026 export. Publications tied at 48 citations are selectively displayed; The Effect of Required Physiatrist Consultation on Surgery Rates for Back Pain (2013) also had 48 citations. Citations per year were calculated using the number of calendar years from publication through 2026, inclusive, based on the citation data exported on 28 May 2026.

**Table 6:** Most frequent standardized keywords in the retrieved dataset.

Standardized Keyword	Number of Publications
Care	18
Shared decision making	17
Decision making	12
Informed consent	12
Health	11
Quality	10
Medical ethics	9
Disability	8
Life	8
Palliative care	8
Stroke	8
Management	7
Competence	6
Impact	6
Inpatient rehabilitation	6
Quality-of-life	6
Resource allocation	6
Adults	5
Brain injury	5
Capacity	5
End of life	5
Outcomes	5
Physicians	5
AIDS	4
Attitudes	4
Autonomy	4
Barriers	4
Burnout	4
Communication	4
Mechanical ventilation	4
Participation	4
People	4
Physical therapy	4
Public health	4
Qualitative research	4

Note: Counts represent the number of unique publications containing each standardized term in either the Author Keywords or Keywords Plus fields, with each term counted once per publication. Keyword normalization was performed according to Supplementary Table S1. The field-defining terms “rehabilitation”, “ethics”, “rehabilitation medicine”, and “physical and rehabilitation medicine” were excluded from thematic displays. Standardized terms occurring in at least four publications were included, and all terms tied at the minimum occurrence threshold were retained. No additional keyword was excluded on the basis of subjective thematic relevance.

**Table 7:** Thematic clusters of the standardized keyword co-occurrence network.

Cluster	Interpretive Theme	Terms, *n*	Principal Standardized Keywords (Publications, *n*)	Within-Cluster Link Weight
**1**	Care, decision-making and rehabilitation access	15	care (18); shared decision making (17); health (11); medical ethics (9); stroke (8); resource allocation (6)	93
**2**	Palliative and participation-related concepts	8	palliative care (8); quality-of-life (6); adults (5); end of life (5); participation (4)	21
**3**	Consent and decision-making capacity	5	informed consent (12); decision making (12); competence (6); capacity (5); outcomes (5)	28
**4**	Disability, quality and attitudes	5	quality (10); life (8); disability (8); attitudes (4); mechanical ventilation (4)	19
**5**	Physicians and AIDS (small cluster)	2	physicians (5); AIDS (4)	2

## Data Availability

Bibliographic records and citation counts were obtained from the Web of Science Core Collection. Redistribution of the original export files is subject to the applicable Clarivate license. Derived data may be requested from the corresponding author.

## Supplementary Materials

The following supporting information is available online at: https://www.mdpi.com/article/10.3390/healthcare14213754/s1, Supplementary Methods S1: Web of Science Core Collection Search Strategy. This document provides the reconstructed search query, field-specific search terms, Boolean operators, database restrictions, and search refinement procedures; Supplementary Table S1: Keyword normalization, term mapping, and exclusion rules applied to Author Keywords and Keywords Plus. The table presents original keyword forms, standardized terms, publication frequencies, and normalization procedures; Supplementary File S1: Bibliographic records included in the primary PM&R ethics-related analysis (*n* = 109). This file provides the bibliographic details and Web of Science accession numbers of the included publications; Supplementary Figure S1: International co-authorship network in PM&R ethics-related publications. Node size represents country-level publication counts, and link width represents the number of internationally co-authored records; Supplementary Figure S2: Temporal profile of frequently occurring standardized keywords. The figure presents the mean publication year of records containing each keyword and the corresponding publication frequency. The analysis is descriptive and does not constitute a formal trend test.
