# Supplementary material for: Ethics in Physical Medicine and Rehabilitation: Global Research Trends and Thematic Structure—A Bibliometric and Science-Mapping Analysis
Source: Healthcare (Basel). 2026 Nov 4;14(21):3754. doi: 10.3390/healthcare14213754 (PMC13763332; doi:10.3390/healthcare14213754)
Supplement: Supplementary file 1 [file healthcare-14-03754-s001.zip › Supplementary File S1.pdf]

Supplementary File S1

Bibliographic records included in the primary PM&R ethics-related analysis (n = 109)

Source and coverage. Web of Science Core Collection export dated 28 May 2026; records published from 1986 through 2025. Items are arranged alphabetically by the first indexed author. Entries retain original bibliographic metadata; unavailable DOI and publication details have not been inferred. Every item includes its unique Web of Science accession number.

Interpretation. The refined bibliographic search initially identified 110 distinct Web of Science records. Following a post hoc title-and-abstract eligibility reassessment, record A024 (Downing, 2011; veterinary rehabilitation) was excluded from the primary analytical dataset, yielding 109 records. Original Appendix A identifiers are retained for traceability; A024 is intentionally absent from the included-record list. Thirty-five flagged records were reviewed by the authors and considered eligible for the human PM&R ethics-related scope. Five potentially overlapping pairs (A005/A006, A039/A040, A041/A042, A094/A095, A105/A106) remain as separately indexed records in the primary analysis; the 1995 records A006, A040, A042, A095, and A106 were excluded only in an additional 104-record sensitivity analysis. Inclusion in the search-defined corpus does not establish that ethics is the primary focus of every record.

A001. Ahadi, T; Mianehsaz, E; Raissi, G; Moraveji, SA; Sharifi, V. (2015). **Professionalism in residents of physical medicine and rehabilitation in Iran.** *JOURNAL OF MEDICAL ETHICS AND HISTORY OF MEDICINE.* 8: Article 3. WoS ID: WOS:000442834100003.

A002. Anderson, CB; Fatone, S; Mañago, MM; Swink, LA; Hager, ER; Kittelson, AJ; Christiansen, CL; Magnusson, DM. (2023). **Improving shared decision-making for prosthetic care: A qualitative needs assessment of prosthetists and new lower-limb prosthesis users.** *PROSTHETICS AND ORTHOTICS INTERNATIONAL.* 47(1): 26–42. DOI: 10.1097/PXR.0000000000000142. WoS ID: WOS:001131172100004.

A003. Arora, A; Chang, P; Asher, A. (2024). **The Role of Rehabilitation for the Dying Cancer Patient.** *CURRENT PHYSICAL MEDICINE AND REHABILITATION REPORTS.* 12(1): 119–125. DOI: 10.1007/s40141-024-00433-x. WoS ID: WOS:001151202300001.

A004. Auerbach, VS; Banja, JD. (1996). **Assessing client competence to participate in rehabilitation decision making.** *NEUROREHABILITATION.* 6(2): 123–132. DOI: 10.1016/1053-8135(96)81032-8. WoS ID: WOS:A1996UE91000004.

A005. BACH, JR; BARNETT, V. (1994). **ETHICAL CONSIDERATIONS IN THE MANAGEMENT OF INDIVIDUALS WITH SEVERE NEUROMUSCULAR DISORDERS.** *AMERICAN JOURNAL OF PHYSICAL MEDICINE & REHABILITATION.* 73(2): 134–140. DOI: 10.1097/00002060-199404000-00012. WoS ID: WOS:A1994NG54200012.

A006. BACH, JR; BARNETT, V. (1995). **ETHICAL CONSIDERATIONS IN THE MANAGEMENT OF INDIVIDUALS WITH SEVERE NEUROMUSCULAR DISORDERS.** *AMERICAN JOURNAL OF PHYSICAL MEDICINE & REHABILITATION.* Issue 1, Supplement S: S34–S40. WoS ID: WOS:A1995QK81900009.

A007. Bang, G; Lee, SJ; Lee, B; Park, M; Park, SY. (2022). **Genetic Testing on Patients with Developmental Delay: A Preliminary Study from the Perspective of Physicians.** *HEALTHCARE.* 10(7): Article 1236. DOI: 10.3390/healthcare10071236. WoS ID: WOS:000834387600001.

A008. Banja, JD; Eisen, A. (2013). **Ethical Perspectives on Knowledge Translation in Rehabilitation.** *ARCHIVES OF PHYSICAL MEDICINE AND REHABILITATION.* 94(1), Supplement 1: S55–S60. DOI: 10.1016/j.apmr.2012.08.218. WoS ID: WOS:000313308400008.

A009. Barradell, AC; Gerlis, C; Houchen-Wolloff, L; Bekker, HL; Robertson, N; Singh, SJ. (2023). **Systematic review of shared decision-making interventions for people living with chronic respiratory diseases.** *BMJ OPEN.* 13(5). DOI: 10.1136/bmjopen-2022-069461. WoS ID: WOS:001001783700018.

A010. Blackmer, J. (2000). **Ethical issues in rehabilitation medicine.** *SCANDINAVIAN JOURNAL OF REHABILITATION MEDICINE.* 32(2): 51–55. DOI: 10.1080/003655000750045541. WoS ID: WOS:000086957100001.

A011. Blackmer, J. (2001). **Tube feeding in stroke patients: A medical and ethical perspective.** *CANADIAN JOURNAL OF NEUROLOGICAL SCIENCES.* 28(2): 101–106. DOI: 10.1017/S0317167100052756. WoS ID: WOS:000168795700002.

A012. Bodenheimer, CF; Roig, RL; Worsowicz, GM; Cifu, DX. (2004). **Geriatric rehabilitation. 5. The societal aspects of disability in the older adult.** *ARCHIVES OF PHYSICAL MEDICINE AND REHABILITATION.* 85(7), Supplement 3: S23–S26. DOI: 10.1016/j.apmr.2004.03.009. WoS ID: WOS:000222679600006.

A013. Bontke, CF. (1997). **Managed care in traumatic brain injury rehabilitation: Physiatrists' concerns and ethical dilemmas.** *JOURNAL OF HEAD TRAUMA REHABILITATION.* 12(1): 37–43. DOI: 10.1097/00001199-199702000-00007. WoS ID: WOS:A1997WG28000007.

A014. Brennan, M. (2013). **Four Butterflies: End of Life Stories of Transition and Transformation.** *PASTORAL PSYCHOLOGY.* 62(2): 139–149. DOI: 10.1007/s11089-012-0477-5. WoS ID: WOS:000212839100003.

A015. Brigham, EJ; Tellers, CA; Rondinelli, R. (2001). **Academic survival through mission-based management.** *AMERICAN JOURNAL OF PHYSICAL MEDICINE & REHABILITATION.* 80(10): 778–785. DOI: 10.1097/00002060-200110000-00013. WoS ID: WOS:000171096500011.

A016. Brooks, FA; Keeney, T; Ritchie, C; Tolchin, DW. (2021). **Rehabilitation for Symptom Management for Patients with Cancer at the End of Life: Current Evidence and Barriers to Care.** *CURRENT PHYSICAL MEDICINE AND REHABILITATION REPORTS.* 9(4): 215–223. DOI: 10.1007/s40141-021-00325-4. WoS ID: WOS:000705812200001.

A017. Caplan, A. (1997). **The ethics of gatekeeping in rehabilitation medicine.** *JOURNAL OF HEAD TRAUMA REHABILITATION.* 12(1): 29–36. DOI: 10.1097/00001199-199702000-00006. WoS ID: WOS:A1997WG28000006.

A018. CAPLAN, AL; CALLAHAN, D; HAAS, J. (1987). **ETHICAL AND POLICY ISSUES IN REHABILITATION-MEDICINE.** *HASTINGS CENTER REPORT.* 17(4): S1–S20. WoS ID: WOS:A1987J790800007.

A019. CAPLAN, AL. (1988). **INFORMED CONSENT AND PROVIDER-PATIENT RELATIONSHIPS IN REHABILITATION-MEDICINE.** *ARCHIVES OF PHYSICAL MEDICINE AND REHABILITATION.* 69(5): 312–317. WoS ID: WOS:A1988N412000002.

A020. Capozzi, LC; Daun, JT; Ester, M; Mosca, S; Langelier, D; Francis, GJ; Chang, E; Mina, DS; Fu, JB; Culos-Reed, SN. (2021). **Physical Activity for Individuals Living with Advanced Cancer: Evidence and Recommendations.** *SEMINARS IN ONCOLOGY NURSING.* 37(4): Article 151170. DOI: 10.1016/j.soncn.2021.151170. WoS ID: WOS:000685539600004.

A021. Catt, JH. (2000). **The language of ethics in clinical practice.** *JOURNAL OF MEDICAL SPEECH-LANGUAGE PATHOLOGY.* 8(3): 137–153. WoS ID: WOS:000089179400003.

A022. Chua, KSG; Ng, YS; Yap, SGM; Bok, CW. (2007). **A brief review of traumatic brain injury rehabilitation.** *ANNALS ACADEMY OF MEDICINE SINGAPORE.* 36(1): 31–42. WoS ID: WOS:000244173500006.

A023. Chuang, YH; Wang, CC; Hsiao, CY; Lu, CY; Wu, JC; Hou, WH. (2024). **Experiences and perspectives related to shared decision-making among outpatients with degenerative joint disease in Taiwan: a qualitative study.** *BMJ OPEN.* 14(2): Article e075693. DOI: 10.1136/bmjopen-2023-075693. WoS ID: WOS:001263656500032.

A025. Elwyn, G; Leavitt, HB; Vesling, M; Jacobs, CR; Lund, L. (2025). **'A return on investment'. How shared decision-making benefits doctors: a key informant interview study.** *BMJ OPEN.* 15(12): Article e107732. DOI: 10.1136/bmjopen-2025-107732. WoS ID: WOS:001649181100001.

A026. Farisco, M; Formisano, R; Gosseries, O; Kato, Y; Koboyashi, S; Laureys, S; Lejeune, N; Martial, C; Matar, A; Morrisey, AM; Schnakers, C; Yakufujiang, M; Yamaki, T; Veeramuthu, V; Zandalasini, M; Zasler, N; Magliacano, A; Estraneo, A. (2024). **International survey on the implementation of the European and American guidelines on disorders of consciousness.** *JOURNAL OF NEUROLOGY.* 271(1): 395–407. DOI: 10.1007/s00415-023-11956-z. WoS ID: WOS:001080680500002.

A027. Finestone, HM; Blackmer, J. (2007). **Refusal to eat, capacity, and ethics in stroke patients: A report of 3 cases.** *ARCHIVES OF PHYSICAL MEDICINE AND REHABILITATION.* 88(11): 1474–1477. DOI: 10.1016/j.apmr.2007.07.018. WoS ID: WOS:000250774400017.

A028. Finestone, HM; Yanni, MM; Dalzell, CJ. (2015). **Patients' recall of diagnostic and treatment information improves with use of the Pain Explanation and Treatment Diagram in an outpatient chronic pain clinic.** *PAIN RESEARCH & MANAGEMENT.* 20(3): 145–151. DOI: 10.1155/2015/897293. WoS ID: WOS:000354577100008.

A029. Fins, JJ. (2019). **Disorders of Consciousness, Past, Present, and Future.** *CAMBRIDGE QUARTERLY OF HEALTHCARE ETHICS.* 28(4): 603–615. DOI: 10.1017/S0963180119000719. WoS ID: WOS:000486222700005.

A030. Flanagan, SR; Whiteson, J; Hall, G; Standaert, CJ. (2017). **Physical Medicine and Rehabilitation Value in Bundled Payment for Total Joint Replacement and Cardiac Surgery: The Rusk Experience.** *PM&R.* 9(11): 1167–1170. DOI: 10.1016/j.pmrj.2017.08.447. WoS ID: WOS:000417113500012.

A031. Fox, J; Haig, AJ; Todey, B; Challa, S. (2013). **The Effect of Required Physiatrist Consultation on Surgery Rates for Back Pain.** *SPINE.* 38(3): E178–E184. DOI: 10.1097/BRS.0b013e31827bf40c. WoS ID: WOS:000333023500007.

A032. Fregni, F; Imamura, M; Chien, HF; Lew, HL; Boggio, P; Kaptchuk, TJ; Riberto, M; Hsing, WT; Battistella, LR; Furlan, A. (2010). **Challenges and Recommendations for Placebo Controls in Randomized Trials in Physical and Rehabilitation Medicine A Report of the International Placebo Symposium Working Group.** *AMERICAN JOURNAL OF PHYSICAL MEDICINE & REHABILITATION*. 89(2): 160–172. DOI: 10.1097/PHM.0b013e3181bc0bbd. WoS ID: WOS:000274117300013.

A033. Friedman, A; Friedman, B; Lutzky, L; Friedman, E; Strous, RD; Treger, I. (2022). **Ethical Issues in Medical Rehabilitation The Dilemma of Who to Admit.** *AMERICAN JOURNAL OF PHYSICAL MEDICINE & REHABILITATION*. 101(12): 1168–1174. DOI: 10.1097/PHM.0000000000001971. WoS ID: WOS:000886610400020.

A034. Friedman, A; Jotkowitz, A; Novack, V; Treger, I. (2023). **Determining ethical criteria for admissions to a rehabilitation unit: a survey of senior rehabilitation physicians.** *INTERNATIONAL JOURNAL OF THERAPY AND REHABILITATION*. 31(8): Article 87. DOI: 10.12968/ijtr.2023.0087. WoS ID: WOS:001338688500001.

A035. Gaber, TAZK. (2006). **Medico-legal and ethical aspects in the management of wandering patients following brain injury: Questionnaire survey.** *DISABILITY AND REHABILITATION*. 28(22): 1413–1416. DOI: 10.1080/09638280600638208. WoS ID: WOS:000241600400007.

A036. Gibbs, RL; Rosen, BS; Lacerte, M. (2013). **Published Research and Physiatric Opinion in Life Care Planning.** *PHYSICAL MEDICINE AND REHABILITATION CLINICS OF NORTH AMERICA*. 24(3): 553–+. DOI: 10.1016/j.pmr.2013.03.002. WoS ID: WOS:000324149800011.

A037. Green, M; Carter, B; Lasky, A. (2016). **Difficult Discharge in Pediatric Rehabilitation Medicine Causing Moral Distress.** *JOURNAL OF SOCIAL WORK IN DISABILITY & REHABILITATION*. 15(1): 42–51. DOI: 10.1080/1536710X.2016.1124253. WoS ID: WOS:000387346900003.

A038. Gutenbrunner, C; Korallus, C; Egen, C; Schiller, J; Sturm, C; Teixido, L; Eckhardt, I; Boekel, A. (2022). **Test of the Rehabilitation Goal Screening (ReGoS) Tool to Support Decision Making and Goal Setting in Physical and Rehabilitation Medicine Practice.** *INTERNATIONAL JOURNAL OF ENVIRONMENTAL RESEARCH AND PUBLIC HEALTH*. 19(23): Article 15562. DOI: 10.3390/ijerph192315562. WoS ID: WOS:000896111300001.

A039. HAAS, J. (1993). **ETHICAL CONSIDERATIONS OF GOAL-SETTING FOR PATIENT-CARE IN REHABILITATION-MEDICINE.** *AMERICAN JOURNAL OF PHYSICAL MEDICINE & REHABILITATION*. 72(4): 228–232. DOI: 10.1097/00002060-199308000-00011. WoS ID: WOS:A1993LU17600011.

A040. HAAS, J. (1995). **ETHICAL CONSIDERATIONS OF GOAL-SETTING FOR PATIENT-CARE IN REHABILITATION-MEDICINE.** *AMERICAN JOURNAL OF PHYSICAL MEDICINE & REHABILITATION*. Issue 1, Supplement S: S16–S20. WoS ID: WOS:A1995QK81900005.

A041. HAAS, JF. (1994). **ETHICAL ISSUES IN PHYSICAL MEDICINE AND REHABILITATION - CONCLUSION TO A SERIES.** *AMERICAN JOURNAL OF PHYSICAL MEDICINE & REHABILITATION*. 73(6): 436–440. DOI: 10.1097/00002060-199411000-00009. WoS ID: WOS:A1994PY51100009.

A042. HAAS, JF. (1995). **ETHICAL ISSUES IN PHYSICAL MEDICINE AND REHABILITATION - CONCLUSION TO A SERIES.** *AMERICAN JOURNAL OF PHYSICAL MEDICINE & REHABILITATION*. Issue 1, Supplement S: S54–S58. WoS ID: WOS:A1995QK81900013.

A043. HAAS, JF; MACKENZIE, CA. (1995). **THE ROLE OF ETHICS IN REHABILITATION-MEDICINE - INTRODUCTION TO A SERIES.** *AMERICAN JOURNAL OF PHYSICAL MEDICINE & REHABILITATION*. Issue 1, Supplement S: S3–S6. WoS ID: WOS:A1995QK81900002.

A044. Hao, XX; Han, L; Zheng, DY; Jin, XZ; Li, CG; Huang, L; Huang, ZH. (2023). **Assessing resource allocation based on workload: a data envelopment analysis study on clinical departments in a class a tertiary public hospital in China.** *BMC HEALTH SERVICES RESEARCH*. 23(1): Article 808. DOI: 10.1186/s12913-023-09803-y. WoS ID: WOS:001037453700002.

A045. Hardart, MKM; Burns, JP; Truog, RD. (2002). **Respiratory support in spinal muscular atrophy type I: A survey of physician practices and attitudes.** *PEDIATRICS*. 110(2): Article e24. DOI: 10.1542/peds.110.2.e24. WoS ID: WOS:000177274800010.

A046. Harris, CA; Muller, JM; Shauver, MJ; Chung, KC. (2018). **Leveraging the Medical Context to Increase Upper Extremity Reconstruction Among Patients With Tetraplegia: A Qualitative Analysis.** *ARCHIVES OF PHYSICAL MEDICINE AND REHABILITATION*. 99(3): 459–467. DOI: 10.1016/j.apmr.2017.06.028. WoS ID: WOS:000427809500005.

A047. Henderson, AW; Turner, AP; Leonard, C; Sayre, G; Suckow, B; Williams, SL; Norvell, DC; Czerniecki, JM. (2023). **Mortality Conversations Between Male Veterans and Their Providers Prior to Dysvascular Lower Extremity Amputation.** *ANNALS OF VASCULAR SURGERY*. 92: 313–322. DOI: 10.1016/j.avsg.2023.01.042. WoS ID: WOS:000986024300001.

A048. Huang, HL; Cheng, SY; Tsai, JS; Su, HY; Lin, YC; Kang, YC; Lee, SY; Chen, YW; Lin, HJ. (2025). **Shared Decision-Making With Otolaryngologists and Palliative Care Specialists in Oral Cavity Cancer.** *JAMA NETWORK OPEN*. 8(12): Article e2548557. DOI: 10.1001/jamanetworkopen.2025.48557. WoS ID: WOS:001638000400008.

A049. Hvidt, NC; Nielsen, KT; Korup, AK; Prinds, C; Hansen, DG; Viftrup, DT; Hvidt, EA; Hammer, ER; Falko, E; Locher, F; Boelsbjerg, HB; Wallin, JA; Thomsen, KF; Schroder, K; Moestrup, L; Nissen, RD; Stewart-Ferrer, S; Stripp, TK; Steenfeldt, VO; Sondergaard, J; Wæhrens, EE. (2020). **What is spiritual care? Professional perspectives on the concept of spiritual care identified through group concept mapping.** *BMJ OPEN*. 10(12): Article e042142. DOI: 10.1136/bmjopen-2020-042142. WoS ID: WOS:000605320900008.

A050. Hwang, R; Lambrechts, S; Liu, H; Saigal, CS; Kwan, L; Cisneros, C; Holly, LT; Duru, OK. (2018). **Decisional Conflict Among Patients Considering Treatment Options for Lumbar Herniated Disc.** *WORLD NEUROSURGERY*. 116: E680–E690. DOI: 10.1016/j.wneu.2018.05.068. WoS ID: WOS:000439498500087.

A051. Jesudason, E. (2024). **Consent with complications in mind.** *JOURNAL OF MEDICAL ETHICS*. 50(11): 758–761. DOI: 10.1136/jme-2023-109731. WoS ID: WOS:001148393200001.

A052. Jesudason, EC. (2025). **The 4D Model: Rehabilitating Unrealistic Medicine.** *BRITISH JOURNAL OF HOSPITAL MEDICINE*. 86(4): Article 845. DOI: 10.12968/hmed.2024.0845. WoS ID: WOS:001477005800010.

A053. Jesus, TS; Papadimitriou, C; Bright, FA; Kayes, NM; Pinho, CS; Cott, CA. (2022). **Person-Centered Rehabilitation Model: Framing the Concept and Practice of Person-Centered Adult Physical Rehabilitation Based on a Scoping Review and Thematic Analysis of the Literature.** *ARCHIVES OF PHYSICAL MEDICINE AND REHABILITATION*. 103(1): 106–120. DOI: 10.1016/j.apmr.2021.05.005. WoS ID: WOS:000735381500015.

A054. Jox, RJ; Kuehlrneyer, K; Klein, AM; Herzog, J; Schaupp, M; Nowak, DA; Koenig, E; Müller, F; Bender, A. (2015). **Diagnosis and Decision Making for Patients With Disorders of Consciousness: A Survey Among Family Members.** *ARCHIVES OF PHYSICAL MEDICINE AND REHABILITATION*. 96(2): 323–330. DOI: 10.1016/j.apmr.2014.09.030. WoS ID: WOS:000348751800020.

A055. Kardes, S; Levack, W; Özkuk, K; Aydin, EA; Karabulut, SS. (2020). **Retractions in Rehabilitation and Sport Sciences Journals: A Systematic Review.** *ARCHIVES OF PHYSICAL MEDICINE AND REHABILITATION*. 101(11): 1980–1990. DOI: 10.1016/j.apmr.2020.03.010. WoS ID: WOS:000582754600017.

A056. Kim, GJ; Gahlot, A; Magsombol, C; Waskiewicz, M; Capasso, N; Van Lew, S; Kim, H; Parnandi, A; Dickson, VV; Goverover, Y. (2024). **Feasibility of a Self-directed Upper Extremity Training Program to Promote Actual Arm Use for Individuals Living in the Community With Chronic Stroke.** *ARCHIVES OF REHABILITATION RESEARCH AND CLINICAL TRANSLATION*. 6(1): Article 100316. DOI: 10.1016/j.arrct.2023.100316. WoS ID: WOS:001208923600001.

A057. Kirschner, KL; Brashler, R; Savage, TA. (2007). **Ashley X.** *AMERICAN JOURNAL OF PHYSICAL MEDICINE & REHABILITATION*. 86(12): 1023–1029. DOI: 10.1097/PHM.0b013e31815b77b2. WoS ID: WOS:000251352600011.

A058. Lam, AKH; Fung, OHY; Kwan, C; Cheung, JPY; Luk, KDK; Chiu, AYY; Descarreaux, M; Szeto, GPY; Wong, AYL. (2022). **The Concerns and Experiences of Patients With Lumbar Spinal Stenosis Regarding Prehabilitation and Recovery After Spine Surgery: A Qualitative Study.** *ARCHIVES OF REHABILITATION RESEARCH AND CLINICAL TRANSLATION*. 4(4): Article 100227. DOI: 10.1016/j.arrct.2022.100227. WoS ID: WOS:001135941400017.

A059. Le Fort, M; Latte, DDD; Perrouin-Verbe, B; Ville, I. (2023). **Organizational ethics in urgent transfers of severely disabled people to intensive care units - a qualitative study.** *DISABILITY AND REHABILITATION*. 45(23): 3852–3860. DOI: 10.1080/09638288.2022.2140847. WoS ID: WOS:000882899100001.

A060. Leonard, C; Sayre, G; Williams, S; Henderson, A; Norvell, D; Turner, AP; Czerniecki, J. (2023). **Perceived shared decision-making among patients undergoing lower-limb amputation and their care teams: A qualitative study.** *PROSTHETICS AND ORTHOTICS INTERNATIONAL*. 47(4): 379–386. DOI: 10.1097/PXR.0000000000000234. WoS ID: WOS:001131215400006.

A061. Maffoni, M; Sommovigo, V; Giardini, A; Paolucci, S; Setti, I. (2020). **Dealing with ethical issues in rehabilitation medicine: The relationship between managerial support and emotional exhaustion is mediated by moral distress and enhanced by positive affectivity and resilience.** *JOURNAL OF NURSING MANAGEMENT*. 28(5): 1114–1125. DOI: 10.1111/jonm.13059. WoS ID: WOS:000542113800001.

A062. Maffoni, M; Sommovigo, V; Giardini, A; Argentero, P; Setti, I. (2021). **THE ITALIAN VERSION OF THE HOSPITAL ETHICAL CLIMATE SURVEY: FIRST PSYCHOMETRIC EVALUATIONS IN A SAMPLE OF HEALTHCARE PROFESSIONALS EMPLOYED IN NEUROREHABILITATION MEDICINE AND PALLIATIVE CARE SPECIALTIES.** *TPM-TESTING PSYCHOMETRICS METHODOLOGY IN APPLIED PSYCHOLOGY*. 28(4): 441–466. DOI: 10.4473/TPM28.4.4. WoS ID: WOS:000755734300004.

A063. Maffoni, M; Sommovigo, V; Giardini, A; Velutti, L; Setti, I. (2022). **Well-Being and Professional Efficacy Among Health Care Professionals: The Role of Resilience Through the Mediation of Ethical Vision of Patient Care and the Moderation of Managerial Support.** *EVALUATION & THE HEALTH PROFESSIONS.* 45(4): 381–396. DOI: 10.1177/01632787211042660. WoS ID: WOS:000697015800001.

A064. Mangin, D; Stephen, G; Bismah, V; Risdon, C. (2016). **Making patient values visible in healthcare: a systematic review of tools to assess patient treatment priorities and preferences in the context of multimorbidity.** *BMJ OPEN.* 6(6): Article e010903. DOI: 10.1136/bmjopen-2015-010903. WoS ID: WOS:000380237100073.

A065. Manhas, KP; Olson, K; Churchill, K; Faris, P; Vohra, S; Wasylak, T. (2020). **Measuring shared decision-making and collaborative goal setting in community rehabilitation: a focused ethnography using cross-sectional surveys in Canada.** *BMJ OPEN.* 10(8): Article e034745. DOI: 10.1136/bmjopen-2019-034745. WoS ID: WOS:000564351500038.

A066. Marois, P; Marois, M; Pouliot-Laforte, A; Vanasse, M; Lambert, J; Ballaz, L. (2016). **Gross Motor Function Measure Evolution Ratio: Use as a Control for Natural Progression in Cerebral Palsy.** *ARCHIVES OF PHYSICAL MEDICINE AND REHABILITATION.* 97(5): 807–814. DOI: 10.1016/j.apmr.2015.07.024. WoS ID: WOS:000375633300020.

A067. Mazlina, M; Julia, PE. (2011). **Attitudes of rehabilitation medicine doctors toward medical ethics in Malaysia.** *SINGAPORE MEDICAL JOURNAL.* 52(6): 421–427. WoS ID: WOS:000292912100016.

A068. Meixner, C; O'Donoghue, CR. (2021). **Access to Care for Persons With Brain Injury: Ethical Frameworks to Promote Health Systems Change.** *JOURNAL OF HEAD TRAUMA REHABILITATION.* 36(1): 72–77. DOI: 10.1097/HTR.0000000000000590. WoS ID: WOS:000604011300013.

A069. Negrini, S; Ceravolo, MG; Ferriero, G. (2022). **Trust/untrust is not the same as true/false. Lessons learned and ethical questions on the application of untrustworthiness scales to judge individuals.** *EUROPEAN JOURNAL OF PHYSICAL AND REHABILITATION MEDICINE.* 58(6): 888–891. DOI: 10.23736/S1973-9087.22.07767-X. WoS ID: WOS:001013360900016.

A070. Nica, AS; Constantinovici, M; Miclaus, R. (2017). **Ethical issues in rehabilitation of the post-traumatic patient.** *ROMANIAN JOURNAL OF LEGAL MEDICINE.* 25(3): 309–313. DOI: 10.4323/rjlm.2017.309. WoS ID: WOS:000417156800016.

A071. Nica, AS; Ghiorghiu, I; Constantinovici, M; Miclaus, RS; Mitoiu, BI. (2019). **Management and ethics issues in rehabilitation and balneoclimatology.** *ROMANIAN JOURNAL OF LEGAL MEDICINE.* 27(2): 195–199. DOI: 10.4323/rjlm.2019.195. WoS ID: WOS:000501873000020.

A072. Niederer, D; Wilke, J; Vogt, L; Banzer, W. (2018). **Return to Play After Injuries: A Survey on the Helpfulness of Various Forms of Assistance in the Shared Decision-Making Process in Semiprofessional Athletes in Germany.** *ARCHIVES OF PHYSICAL MEDICINE AND REHABILITATION.* 99(4): 690–698. DOI: 10.1016/j.apmr.2017.10.019. WoS ID: WOS:000428965300012.

A073. Osterthun, R; Sunnerhagen, KS; Stam, HJ; Kiekens, C. (2023). **END-OF-LIFE DECISIONS AND INVOLVEMENT OF PHYSICAL AND REHABILITATION MEDICINE PHYSICIANS IN EUROPE.** *JOURNAL OF REHABILITATION MEDICINE.* 55: Article jrm5575. DOI: 10.2340/jrm.v55.5575. WoS ID: WOS:001025258400004.

A074. Papadimitriou, C; Clayman, ML; Mallinson, T; Weaver, JA; Guernon, A; Meehan, AJ; Kot, T; Ford, P; Ideishi, R; Prather, C; van der Wees, P. (2024). **A New Process Model for Relationship-Centred Shared Decision-Making in Physical Medicine and Rehabilitation Settings.** *HEALTH EXPECTATIONS.* 27(4): Article e14162. DOI: 10.1111/hex.14162. WoS ID: WOS:001290221500001.

A075. Paramasivam, A; Jaiswal, A; Minhas, R; Wittich, W; Spruyt-Rocks, R. (2021). **Informed Consent or Assent Strategies for Research With Individuals With Deafblindness or Dual Sensory Impairment: A Scoping Review.** *ARCHIVES OF REHABILITATION RESEARCH AND CLINICAL TRANSLATION.* 3(2): Article 100115. DOI: 10.1016/j.arrct.2021.100115. WoS ID: WOS:001135941600018.

A076. Priebe, MM; Chiodo, AE; Scelza, WM; Kirshblum, SC; Wuermser, LA; Ho, CH. (2007). **Spinal cord injury medicine. 6. Economic and societal issues in spinal cord injury.** *ARCHIVES OF PHYSICAL MEDICINE AND REHABILITATION.* 88(3), Supplement 1: S84–S88. DOI: 10.1016/j.apmr.2006.12.005. WoS ID: WOS:000244706500017.

A077. PURTILO, RB. (1986). **ETHICAL ISSUES IN THE TREATMENT OF CHRONIC VENTILATOR-DEPENDENT PATIENTS.** *ARCHIVES OF PHYSICAL MEDICINE AND REHABILITATION.* 67(10): 718–721. DOI: 10.1016/0003-9993(86)90002-X. WoS ID: WOS:A1986E336000002.

A078. Rajasekaran, S; Shan, RLP; Finnoff, JT. (2014). **Honorary Authorship: Frequency and Associated Factors in Physical Medicine and Rehabilitation Research Articles.** *ARCHIVES OF PHYSICAL MEDICINE AND REHABILITATION.* 95(3): 418–428. DOI: 10.1016/j.apmr.2013.09.024. WoS ID: WOS:000332055600002.

A079. Raymond, MH; Demers, L; Feldman, DE. (2018). **Differences in Waiting List Prioritization Preferences of Occupational Therapists, Elderly People, and Persons With Disabilities: A Discrete Choice Experiment.** *ARCHIVES OF PHYSICAL MEDICINE AND REHABILITATION.* 99(1): 35–42. DOI: 10.1016/j.apmr.2017.06.031. WoS ID: WOS:000419421600005.

A080. Ringstad, O. (2010). **Interviewing patients and practitioners working together in teams. A multi-layered puzzle: putting the pieces together.** *MEDICINE HEALTH CARE AND PHILOSOPHY.* 13(3): 193–202. DOI: 10.1007/s11019-010-9243-6. WoS ID: WOS:000280845900002.

A081. Rogge, A; Witt, VD; Valdueza, JM; Borzikowsky, C; Buyx, A. (2019). **Experience in Rehabilitation Medicine Affects Prognosis and End-of-Life Decision-Making of Neurologists: A Case-Based Survey.** *NEUROCRITICAL CARE.* 31(1): 125–134. DOI: 10.1007/s12028-018-0661-2. WoS ID: WOS:000474217800018.

A082. Rondinelli, RD; Eskay-Auerbach, M. (2019). **Healthcare Provider Issues and Perspective Impairment Ratings and Disability Determinations.** *PHYSICAL MEDICINE AND REHABILITATION CLINICS OF NORTH AMERICA.* 30(3): 511–+. DOI: 10.1016/j.pmr.2019.04.001. WoS ID: WOS:000477686300004.

A083. Schönhofer, B; Geiseler, J; Dellweg, D; Fuchs, H; Moerer, O; Weber-Carstens, S; Westhoff, M; Windisch, W. (2021). **Prolonged Weaning: S2k Guideline Published by the German Respiratory Society.** *RESPIRATION.* 99(11): 982–1083. DOI: 10.1159/000510085. WoS ID: WOS:000600744400001.

A084. SCOFIELD, GR. (1993). **ETHICAL CONSIDERATIONS IN REHABILITATION-MEDICINE.** *ARCHIVES OF PHYSICAL MEDICINE AND REHABILITATION.* Issue 4: 341–346. WoS ID: WOS:A1993KX39800001.

A085. Shiggins, C; Ryan, B; O'Halloran, R; Power, E; Bernhardt, J; Lindley, RI; McGurk, G; Hankey, GJ; Rose, ML. (2022). **Towards the Consistent Inclusion of People With Aphasia in Stroke Research Irrespective of Discipline.** *ARCHIVES OF PHYSICAL MEDICINE AND REHABILITATION.* 103(11): 2256–2263. DOI: 10.1016/j.apmr.2022.07.004. WoS ID: WOS:000886977800020.

A086. Shojaei, A; Ghane, B. (2016). **Physical Therapy Ethical Challengesin Iran, A Qualitative Study.** *INTERNATIONAL JOURNAL OF ADVANCED BIOTECHNOLOGY AND RESEARCH.* 7: 645–652. WoS ID: WOS:000402909100079.

A087. Silver, JK; Cuccurullo, S; Weiss, LD; Visco, C; Oh-Park, M; Karimi, DP; Frontera, WR; Fleming, TK; Bosques, G; Bhatnagar, S; Ambrose, AF; Nguyen, VQC. (2020). **The Vital Role of Professionalism in Physical Medicine and Rehabilitation.** *AMERICAN JOURNAL OF PHYSICAL MEDICINE & REHABILITATION.* 99(4): 273–277. DOI: 10.1097/PHM.0000000000001322. WoS ID: WOS:000526401900009.

A088. Sliwa, JA; McPeak, L; Gittler, M; Bodenheimer, C; King, J; Bowen, J. (2002). **Clinical ethics in rehabilitation medicine: Core objectives and algorithm for resident education.** *AMERICAN JOURNAL OF PHYSICAL MEDICINE & REHABILITATION.* 81(9): 708–717. DOI: 10.1097/00002060-200209000-00012. WoS ID: WOS:000177661000010.

A089. Smeltz, L; Carpenter, S; Benedetto, L; Newcomb, N; Rubenstein, D; King, T; Lunsford, C; Shaw, T; Dewaters, AL. (2024). **Introduction to Disability and Antiableist Health Care.** *AMERICAN JOURNAL OF PHYSICAL MEDICINE & REHABILITATION.* 103(5): e54–e57. DOI: 10.1097/PHM.0000000000002399. WoS ID: WOS:001205416600006.

A090. Spill, GR; Hlubocky, FJ; Daugherty, CK. (2012). **Oncologists' and Physiatrists' Attitudes Regarding Rehabilitation for Patients With Advanced Cancer.** *PM&R.* 4(2): 96–108. DOI: 10.1016/j.pmrj.2011.08.539. WoS ID: WOS:000305438300002.

A091. Stineman, MG; Ross, RN; Williams, SV; Goin, JE; Granger, CV. (2000). **A functional diagnostic complexity index for rehabilitation medicine: Measuring the influence of many diagnoses on functional independence and resource use.** *ARCHIVES OF PHYSICAL MEDICINE AND REHABILITATION.* 81(5): 549–557. DOI: 10.1053/mr.2000.5977. WoS ID: WOS:000086867800002.

A092. Stineman, MG; Musick, DW. (2001). **Protection of human subjects with disability: Guidelines for research.** *ARCHIVES OF PHYSICAL MEDICINE AND REHABILITATION.* 82(12), Supplement 2: S9–S14. DOI: 10.1053/apmr.2001.31002. WoS ID: WOS:000173270100003.

A093. Stout, NL; Silver, JK; Raj, VS; Rowland, J; Gerber, L; Cheville, A; Ness, KK; Radomski, M; Nitkin, R; Stubblefield, MD; Morris, GS; Acevedo, A; Brandon, Z; Braveman, B; Cunningham, S; Gilchrist, L; Jones, L; Padgett, L; Wolf, T; Winters-Stone, K; Campbell, G; Hendricks, J; Perkin, K; Chan, L. (2016). **Toward a National Initiative in Cancer Rehabilitation: Recommendations From a Subject Matter Expert Group.** *ARCHIVES OF PHYSICAL MEDICINE AND REHABILITATION.* 97(11): 2006–2015. DOI: 10.1016/j.apmr.2016.05.002. WoS ID: WOS:000387634500023.

A094. STRAX, TE. (1994). **ETHICAL ISSUES OF TREATING PATIENTS WITH AIDS IN A REHABILITATION SETTING.** *AMERICAN JOURNAL OF PHYSICAL MEDICINE & REHABILITATION.* 73(4): 293–295. DOI: 10.1097/00002060-199407000-00013. WoS ID: WOS:A1994PB25400013.

A095. STRAX, TE. (1995). **ETHICAL ISSUES OF TREATING PATIENTS WITH AIDS IN A REHABILITATION SETTING.** *AMERICAN JOURNAL OF PHYSICAL MEDICINE & REHABILITATION.* Issue 1, Supplement S: S49–S51. WoS ID: WOS:A1995QK81900011.

A096. Takashi, N; McCarthy, MJ; Suzuki, R; Ogahara, K; Ono-Kihara, M; Kihara, M; Nakayama, T. (2021). **Association of patient quality of life with the degree of agreement in the perceptions of patient disability within the stroke patient-rehabilitation therapist dyad: a cross-sectional study in postdischarge rehabilitation setting.** *BMJ OPEN.* 11(5): Article e043824. DOI: 10.1136/bmjopen-2020-043824. WoS ID: WOS:000713844400040.

A097. Tamburrini, G. (2009). **Brain to Computer Communication: Ethical Perspectives on Interaction Models.** *NEUROETHICS.* 2(3): 137–149. DOI: 10.1007/s12152-009-9040-1. WoS ID: WOS:000284870000003.

A098. Tammany, JE; O'Connell, JK; Allen, BS; Brismee, JM. (2019). **Are Productivity Goals in Rehabilitation Practice Associated With Unethical Behaviors?.** *ARCHIVES OF REHABILITATION RESEARCH AND CLINICAL TRANSLATION.* 1(1-2): Article 100002. DOI: 10.1016/j.arrct.2019.100002. WoS ID: WOS:001134992900001.

A099. Taso, M; Sommernes, JH; Kolstad, F; Sundseth, J; Bjorland, S; Pripp, AH; Zwart, JA; Brox, JI. (2020). **A randomised controlled trial comparing the effectiveness of surgical and nonsurgical treatment for cervical radiculopathy.** *BMC MUSCULOSKELETAL DISORDERS.* 21(1). DOI: 10.1186/s12891-020-3188-6. WoS ID: WOS:000521125200003.

A100. Triebel, KL; Martin, RC; Novack, TA; Dreer, LE; Turner, C; Kennedy, R; Marson, DC. (2014). **Recovery Over 6 Months of Medical Decision-Making Capacity After Traumatic Brain Injury.** *ARCHIVES OF PHYSICAL MEDICINE AND REHABILITATION.* 95(12): 2296–2303. DOI: 10.1016/j.apmr.2014.07.413. WoS ID: WOS:000345954000009.

A101. Tsimba, V. (2018). **Ethical issues in care of children with multiple disabilities.** *MEDECINE PALLIATIVE.* 17(5): 293–299. DOI: 10.1016/j.medpal.2018.03.010. WoS ID: WOS:000449659100011.

A102. van Randwijk, CB; Opsahl, T; Hvidt, EA; Stripp, TK; Bjerrum, L; Herrstedt, J; Sondergaard, J; Hvidt, NC. (2021). **Similarities and Differences between Danish and American Physicians' Religious Characteristics and Clinical Communication: Two Cross-Sectional Surveys.** *RELIGIONS.* 12(2): Article 116. DOI: 10.3390/rel12020116. WoS ID: WOS:000623117600001.

A103. van Til, JA; Drossaert, CHC; Punter, RA; Ijzerman, MJ. (2010). **THE POTENTIAL FOR SHARED DECISION-MAKING AND DECISION AIDS IN REHABILITATION MEDICINE.** *JOURNAL OF REHABILITATION MEDICINE.* 42(6): 598–604. DOI: 10.2340/16501977-0549. WoS ID: WOS:000279001000014.

A104. Vanti, C; Pillastrini, P; Monticone, M; Ceron, D; Bonetti, F; Piccarreta, R; Guccione, A; Violante, FS. (2014). **The Italian version of the Physical Therapy Patient Satisfaction Questionnaire - [PTPSQ-I(15)]: psychometric properties in a sample of inpatients.** *BMC MUSCULOSKELETAL DISORDERS.* 15: Article 135. DOI: 10.1186/1471-2474-15-135. WoS ID: WOS:000335191400002.

A105. VENESY, BA. (1994). **A CLINICIAN GUIDE TO DECISION-MAKING CAPACITY AND ETHICALLY SOUND MEDICAL DECISIONS.** *AMERICAN JOURNAL OF PHYSICAL MEDICINE & REHABILITATION.* 73(3): 219–226. WoS ID: WOS:A1994NR93700014.

A106. VENESY, BA. (1995). **A CLINICIAN GUIDE TO DECISION-MAKING CAPACITY AND ETHICALLY SOUND MEDICAL DECISIONS.** *AMERICAN JOURNAL OF PHYSICAL MEDICINE & REHABILITATION.* Issue 1, Supplement S: S41–S48. WoS ID: WOS:A1995QK81900010.

A107. Wagner, AK; Boninger, ML; Levy, C; Chan, L; Gater, D; Kirby, RL. (2003). **Issues in physical medicine and rehabilitation.** *AMERICAN JOURNAL OF PHYSICAL MEDICINE & REHABILITATION.* 82(10): 790–802. DOI: 10.1097/01.PHM.0000087607.28091.B7. WoS ID: WOS:000185457900009.

A108. Warner, G; Hoenig, H; Montez, M; Wang, F; Rosen, A. (2004). **Evaluating diagnosis-based risk-adjustment methods in a population with spinal cord dysfunction.** *ARCHIVES OF PHYSICAL MEDICINE AND REHABILITATION.* 85(2): 218–226. DOI: 10.1016/S0003-9993(03)00768-8. WoS ID: WOS:000188830400006.

A109. Yamaga, T; Asano, K; Ikeya, M; Nakanishi, K. (2021). **Successful occupational therapy at end of life for a patient with prostate sarcoma.** *BMJ CASE REPORTS.* 14(6): Article e242056. DOI: 10.1136/bcr-2021-242056. WoS ID: WOS:000665126300014.

A110. Yanamadala, V; Kim, Y; Buchlak, QD; Wright, AK; Babington, J; Friedman, A; Mecklenburg, RS; Farrokhi, F; Leveque, JC; Sethi, RK. (2017). **Multidisciplinary Evaluation Leads to the Decreased Utilization of Lumbar Spine Fusion.** *SPINE.* 42(17): E1016–E1023. DOI: 10.1097/BRS.0000000000002065. WoS ID: WOS:000410430300004.
