# Supplementary material for: Ethics in Physical Medicine and Rehabilitation: Global Research Trends and Thematic Structure—A Bibliometric and Science-Mapping Analysis
Source: Healthcare (Basel). 2026 Nov 4;14(21):3754. doi: 10.3390/healthcare14213754 (PMC13763332; doi:10.3390/healthcare14213754)
Supplement: Supplementary file 1 [file healthcare-14-03754-s001.zip › Supplementary Methods S1.pdf]

Supplementary Methods S1. Web of Science Core Collection Search Strategy

Bibliographic records were retrieved from the Web of Science Core Collection (WoSCC). The archived Web of Science Core Collection bibliographic export is dated 28 May 2026. This date represents the documented export date; the original search execution date could not be independently verified. The following indexes were included: Science Citation Index Expanded (SCI-EXPANDED), Social Sciences Citation Index (SSCI), Emerging Sources Citation Index (ESCI), and Arts & Humanities Citation Index (AHCI). Records indexed only in the Conference Proceedings Citation Index-Science (CPCI-S) or Book Citation Index-Science (BKCI-S) were excluded. No language restriction was applied. Eligible document types were Article and Review. Publications from 2026 were excluded; therefore, the final analytical dataset covered publications through 2025. The refined search initially yielded 110 records. Following post hoc eligibility reassessment, one out-of-scope veterinary publication (A024) was excluded, leaving 109 records for the primary bibliometric analysis.

The initial exploratory search used broad Topic-field retrieval for both the Physical Medicine and Rehabilitation (PM&R) and ethics concept blocks. Because this approach retrieved a substantial number of study protocols containing generic ethics statements rather than publications substantively focused on ethical issues, the final strategy was refined. In the final strategy, PM&R-related terminology remained in the Topic field, whereas ethics-related terminology was required to occur in the Title, Author Keywords, or Keywords Plus fields. Records containing protocol-related phrases were excluded using the Topic field.

The executable query presented below was retrospectively reconstructed from the documented field-level search strategy. The archived 110-record dataset was examined for consistency with the reconstructed PM&R- and ethics-related search concepts and available bibliographic restrictions. This assessment supported the internal consistency of the included records but did not constitute an independent reproduction of the historical Web of Science search. Because the original saved search-history string and a reproducible execution log were unavailable, exact equivalence between the reconstructed query and the original retrieval process could not be established.

Final executable Web of Science query

```
TS=(
"physical medicine and rehabilitation"
OR "physical and rehabilitation medicine"
OR "rehabilitation medicine"
OR physiatry
OR physiatrist*
OR "rehabilitation physician*"
)
AND
(
TI=(
ethic*
OR bioethic*
OR "clinical ethics"
OR "medical ethics"
OR "research ethics"
OR "ethical issue*"
OR "ethical dilemma*"
```

OR "moral distress"
OR "professional ethics"
OR "informed consent"
OR "patient autonomy"
OR "shared decision making"
OR "decision-making capacity"
OR "patient right*"
OR "disability right*"
OR ableism
OR antiableis*
OR "resource allocation"
OR "distributive justice"
OR "end-of-life"
OR "end of life"
OR confidentiality
OR futility
)
OR
AK=(
ethic*
OR bioethic*
OR "clinical ethics"
OR "medical ethics"
OR "research ethics"
OR "ethical issue*"
OR "ethical dilemma*"
OR "moral distress"
OR "professional ethics"
OR "informed consent"
OR "patient autonomy"
OR "shared decision making"
OR "decision-making capacity"
OR "patient right*"
OR "disability right*"
OR ableism
OR antiableis*
OR "resource allocation"
OR "distributive justice"
OR "end-of-life"
OR "end of life"
OR confidentiality
OR futility
)
OR
KP=(
ethic*
OR bioethic*
OR "clinical ethics"
OR "medical ethics"
OR "research ethics"
OR "ethical issue*"
OR "ethical dilemma*"
OR "moral distress"
OR "professional ethics"
OR "informed consent"

```
OR "patient autonomy"
OR "shared decision making"
OR "decision-making capacity"
OR "patient right*"
OR "disability right*"
OR ableism
OR antiableis*
OR "resource allocation"
OR "distributive justice"
OR "end-of-life"
OR "end of life"
OR confidentiality
OR futility
)
)
NOT TS=(
"ethics and dissemination"
OR "study protocol"
OR "trial protocol"
OR "protocol for"
)
```

Preliminary broad-search logic

The same PM&R concept block and ethics concept block were initially searched using the Topic field. This preliminary search was exploratory and was used to evaluate retrieval specificity; it was not the dataset used for bibliometric analyses. The broad search yielded 1,056 records before year, document-type, and index restrictions. After exclusion of 2026 records and restriction to eligible document types, 918 records remained; exclusion of records indexed only in CPCI-S or BKCI-S resulted in an intermediate dataset of 917 records. The ethics concept block was subsequently restricted to Title, Author Keywords, and Keywords Plus as described above, yielding the final 110-record dataset after protocol-related exclusions.

Notes

• TS = Topic; TI = Title; AK = Author Keywords; KP = Keywords Plus.

• The original Web of Science search-history string was not preserved as a separate archived file. Therefore, the query is presented as a retrospective reconstruction based on the documented field-level search logic. Consistency checks were performed using the archived final dataset; however, these checks should not be interpreted as independent verification that the reconstructed query would reproduce the complete historical retrieval set.

• The final bibliographic export was saved in the “Full Record and Cited References” format.
