# Supplementary material for: Ethics in Physical Medicine and Rehabilitation: Global Research Trends and Thematic Structure—A Bibliometric and Science-Mapping Analysis
Source: Healthcare (Basel). 2026 Nov 4;14(21):3754. doi: 10.3390/healthcare14213754 (PMC13763332; doi:10.3390/healthcare14213754)
Supplement: Supplementary file 1 [file healthcare-14-03754-s001.zip › Supplementary Table S1.pdf]

Supplementary Table S1. Keyword normalization, term mapping, and exclusion rules applied to Author Keywords and Keywords Plus

Original term in WoS data	n before mapping*	Standardized term	n after mapping†	Action
shared decision-making	14	shared decision making	17	Hyphenation variant merged
shared decision making	3	shared decision making	17	Retained as standardized form
decision-making	4	decision making	12	Hyphenation variant merged
decision making	8	decision making	12	Retained as standardized form
informed-consent	3	informed consent	**12**	Hyphenation variant merged
informed consent	9	informed consent	**12**	Retained as standardized form
medical-ethics	1	medical ethics	**9**	Hyphenation variant merged
medical ethics	8	medical ethics	**9**	Retained as standardized form
end-of-life	1	end of life	5	Hyphenation variant merged
end of life	4	end of life	5	Retained as standardized form
brain-injury	2	brain injury	**5**	Hyphenation variant merged
brain injury	4	brain injury	**5**	Retained as standardized form
rehabilitation-medicine	1	rehabilitation medicine	15	Hyphenation variant merged
rehabilitation medicine	14	rehabilitation medicine	15	Retained as standardized form
low-back-pain	3	low back pain	3	Hyphenation variant merged
low-back pain	1	low back pain	3	Hyphenation variant merged
low back pain	1	low back pain	3	Retained as standardized form
physical-therapy	3	physical therapy	4	Hyphenation variant merged
physical therapy	2	physical therapy	4	Retained as standardized form
ethical-issues	2	ethical issues	3	Hyphenation variant merged
ethical issues	1	ethical issues	3	Retained as standardized form
spinal-cord-injury	1	spinal cord injury	3	Hyphenation variant merged
spinal cord injury	2	spinal cord injury	3	Retained as standardized form
head-injury	2	head injury	2	Hyphenation variant merged
head injury	1	head injury	2	Retained as standardized form
occupational-therapy	1	occupational therapy	3	Hyphenation variant merged
occupational therapy	2	occupational therapy	3	Retained as standardized form
primary-care	1	primary care	2	Hyphenation variant merged
primary care	1	primary care	2	Retained as standardized form
phy-sical and rehabilitation medicine	1	physical and rehabilitation medicine	5	Obvious typographic/hyphenation artifact corrected
physical and rehabilitation medicine	4	physical and rehabilitation medicine	5	Retained as standardized form

** n before mapping: records containing the original label.*

† n after mapping: unique records containing the standardized term after merging; records are counted once even when both variants occur in the same publication.

Keyword preprocessing was conducted at the publication level. Author Keywords and Keywords Plus were extracted separately before being combined for the primary analysis. Repeated occurrences of the same standardized term within a publication were counted only once. Hyphenation and spelling variants were normalized according to the mapping rules presented above. Conceptually distinct terms, including "competence", "capacity", and "decision making", were not merged. The field-defining terms "rehabilitation", "ethics", "rehabilitation medicine", and "physical and rehabilitation medicine" were excluded from thematic displays. No further terms were excluded solely because of their perceived thematic importance or relevance.
